# NIR‐II Responsive Multifunctional Scaffold Enabling “Kill‐Modulation‐Build” Synergistic Therapy for Infectious Bone Defects

**DOI:** 10.1002/advs.202508948

**Published:** 2025-10-07

**Authors:** Qifei Yang, Shu Lou, Yuan Zhang, Zhurun Fang, Changyue Xing, Wentao Wang, Minxuan Han, Zhendong Wang, Ben Zhong Tang, Ming Zhang

**Affiliations:** ^1^ The Affiliated Stomatological Hospital of Nanjing Medical University. State Key Laboratory Cultivation Base of Research Prevention and Treatment for Oral Diseases. Jiangsu Province Engineering Research Center of Stomatological Translational Medicine Nanjing Medical University Nanjing 210029 China; ^2^ College of Science Nanjing Forestry University Nanjing 210037 China; ^3^ School of Science and Engineering Shenzhen Institute of Aggregate Science and Technology The Chinese University of Hong Kong Shenzhen 518172 China

**Keywords:** dynamic immunomodulation, infectious bone regeneration, nanofibrous scaffold, photothermal antibacterial

## Abstract

Infectious bone defects present dual challenges: eliminating bacteria and regenerating tissue, which are further complicated by biofilm formation, antibiotic resistance, and chronic inflammation. Here, a near‐infrared‐II (NIR‐II) responsive fibrous scaffold (AIE NPs@BP NSs@FSs) that employs a sequential “Kill–Modulation–Build” therapeutic strategy—referring to infection clearance, immune regulation, and bone repair is developed. Upon NIR‐II irradiation, aggregation‐induced emission nanoparticles (AIE NPs) produce localized hyperthermia, which eliminates bacteria and biofilms, offering a non‐antibiotic solution that reduces the risk of resistance development. Moreover, black phosphorus nanosheets (BP NSs) scavenge reactive oxygen species (ROS) and reprogram macrophages toward a pro‐regenerative M2 phenotype. This immunomodulatory shift enhances endothelial migration, neovascularization, and osteogenic differentiation of bone marrow stem cells. With excellent biocompatibility and biomimetic architecture, the scaffold supports coordinated antibacterial, immunoregulatory, and osteoinductive responses. This work provides a promising platform for treating complex infected bone defects by combining photothermal therapy with immune and regenerative modulation.

## Graphical abstract

A novel multifunctional scaffold with photothermal effects is developed for infected bone defect repair. It facilitates angiogenesis and osteogenesis while eliminating bacteria through hyperthermia. Additionally, the scaffold promotes macrophage polarization from M1 to M2 via ROS scavenging, enhancing anti‐inflammatory activity. This study demonstrates the scaffold’s dual therapeutic role in antibacterial action and tissue regeneration, offering a promising strategy for treating infected bone defects.

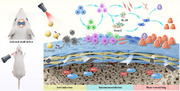

## 1 Introduction

Infected bone defects caused by microbial contamination after severe trauma or surgical procedures remain a major clinical challenge. These cases often require repeated debridement and long‐term systemic antibiotics.^[^ ^1^ ^]^ Bone healing involves three biological phases: inflammation, repair, and remodeling. However, bacterial colonization and biofilm formation in infected bone defects greatly hinder this process.^[^ ^2^ ^]^ Overuse of antibiotics has accelerated the development of drug‐resistant bacteria. Additionally, implanted materials can promote bacterial adhesion, leading to frequent recurrence of osteomyelitis.^[^ ^3^ ^]^ The microenvironment of infected bone is characterized by excessive reactive oxygen species (ROS) and ongoing inflammation, both of which hinder bone regeneration.^[^ ^4^ ^]^ Elevated ROS levels cause osteoblast apoptosis, stimulate osteoclast activity, suppress new blood vessel formation, and prolong inflammation, collectively impeding bone healing.^[^ ^5^, ^6^, ^7^ ^]^ During early inflammation, macrophages shift toward a pro‐inflammatory M1 phenotype, releasing cytokines that increase bone resorption and tissue damage.^[^ ^8^ ^]^ Conversely, M2 macrophages support immune resolution and bone formation, aiding the transition to the repair phase.^[^ ^9^, ^10^ ^]^ Therefore, controlling macrophage polarization is vital for resolving inflammation and restoring bone balance. Given these issues, multifunctional biomaterials with combined antibacterial, anti‐inflammatory, and osteoinductive properties are urgently needed.

Compared to conventional anti‐infective therapies, photothermal therapy (PTT) offers distinct advantages as a non‐invasive, non‐antibiotic strategy, including high selectivity, effective bacterial eradication, minimal risk of resistance development, and controllable treatment parameters.^[^ ^11^, ^12^ ^]^ PTT utilizes photothermal agents (PTAs) to generate localized hyperthermia under near‐infrared (NIR) laser irradiation and has emerged as a promising antibacterial approach.^[^ ^13^ ^]^ However, traditional PTAs operating in the first NIR window (NIR‐I, 700–900 nm) face limitations such as low photothermal conversion efficiency, shallow tissue penetration due to scattering and absorption, and suboptimal stability and biocompatibility.^[^ ^14^, ^15^ ^]^ Common NIR‐I PTAs, including gold nanorods and copper sulfide nanoparticles, suffer from poor biodegradability or potential toxicity.^[^ ^16^ ^]^ Although organic small‐molecule PTAs offer improved biocompatibility, they often exhibit inadequate performance under NIR‐II (1000–1700 nm) irradiation. The emergence of aggregation‐induced emission luminogens (AIEgens) has provided a promising solution. AIEgen‐based PTAs designed for NIR‐II display enhanced photothermal effects due to aggregation, tunable absorption profiles, and excellent biocompatibility.^[^ ^17^, ^18^ ^]^ Therefore, engineering advanced AIEgens as NIR‐II‐responsive PTAs holds significant promise for achieving efficient and safe PTT in clinical settings.

In addition to controlling bacterial infections, regulating excessive reactive oxygen species (ROS) and the inflammatory immune microenvironment is equally critical for effective bone regeneration. Recently, 2D black phosphorus (BP) has attracted considerable attention in the biomedical field due to its outstanding biocompatibility and biodegradability.^[^ ^19^ ^]^ Its degradation products (PO_4_^3−^) play vital roles in biomineralization and osteogenesis.^[^ ^20^, ^21^, ^22^ ^]^ Notably, BP exhibits strong ROS scavenging ability and promotes M2 macrophage polarization, thus alleviating oxidative stress and inflammation while supporting tissue regeneration.^[^ ^23^, ^24^, ^25^ ^]^ Although BP has been reported to possess mild photothermal responsiveness under NIR‐II irradiation, its conversion efficiency is relatively low and insufficient to achieve bactericidal temperatures. Therefore, in this study, BP is primarily utilized for its bioactive and immunoregulatory roles.^[^ ^26^, ^27^, ^28^, ^29^ ^]^ Based on these properties, integrating BP into fibrous scaffolds offers a promising strategy to synergize mechanical support with biological regulation. Such scaffolds emulate the structural architecture of native collagen fibers, providing a highly porous network that enhances cellular infiltration, adhesion, and ultimately bone tissue regeneration.^[^ ^30^, ^31^, ^32^, ^33^, ^34^, ^35^ ^]^

**Figure 1:**
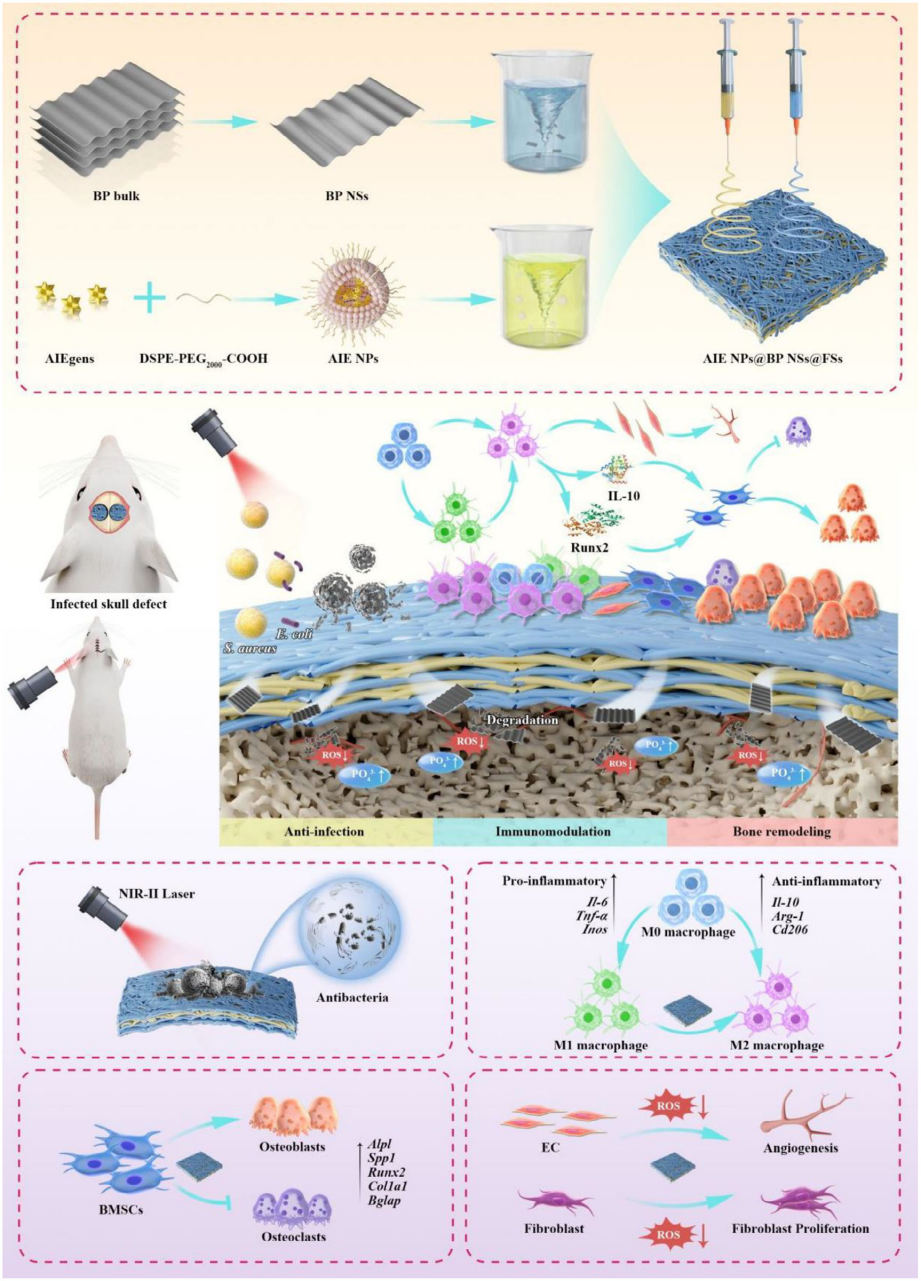
Schematic illustration of the fabrication and therapeutic mechanism of AIE NPs@BP NSs@FSs for treating infected cranial bone defects in rats. AIE NPs and BP NSs are co‐loaded into PLGA fibers via electrospinning to form a multifunctional scaffold. Under NIR‐II irradiation, AIE NPs generate localized heat to eliminate bacteria and biofilms, while BP NSs scavenge ROS and modulate immunity by promoting M2 macrophage polarization. The improved immune microenvironment enhances angiogenesis, fibroblast proliferation, and osteogenic differentiation of BMSCs, thereby facilitating bone regeneration and remodeling.

Here, we developed a NIR‐II responsive fibrous scaffold (AIE NPs@BP NSs@FSs) that combines three sequential therapeutic functions—“Kill–Modulation–Build,” meaning infection eradication, immune modulation, and bone regeneration, respectively—for treating bacteria‐infected bone defects. Considering the connection between infection, inflammation, and tissue regeneration in such defects, our design highlights the coordinated action of antibacterial activity, immune modulation, and reparative signaling to restore the healing process step by step. Our results showed that the multifunctional scaffold effectively eliminated bacterial infections under NIR‐II laser irradiation, reprogrammed the immune environment by increasing anti‐inflammatory markers and decreasing pro‐inflammatory cytokines in vitro. The scaffold promoted blood vessel formation, supported new bone growth, and successfully repaired infected cranial defects in a rat model. This study suggests that AIE NPs@BP NSs@FSs have strong potential as a multifunctional therapeutic platform for treating bacteria‐infected bone defects (**Figure** **1**).

## 2 Results and Discussion

### 2.1 Fabrication and Characterization of AIE NPs@BP NSs@FSs

**Figure 2:**
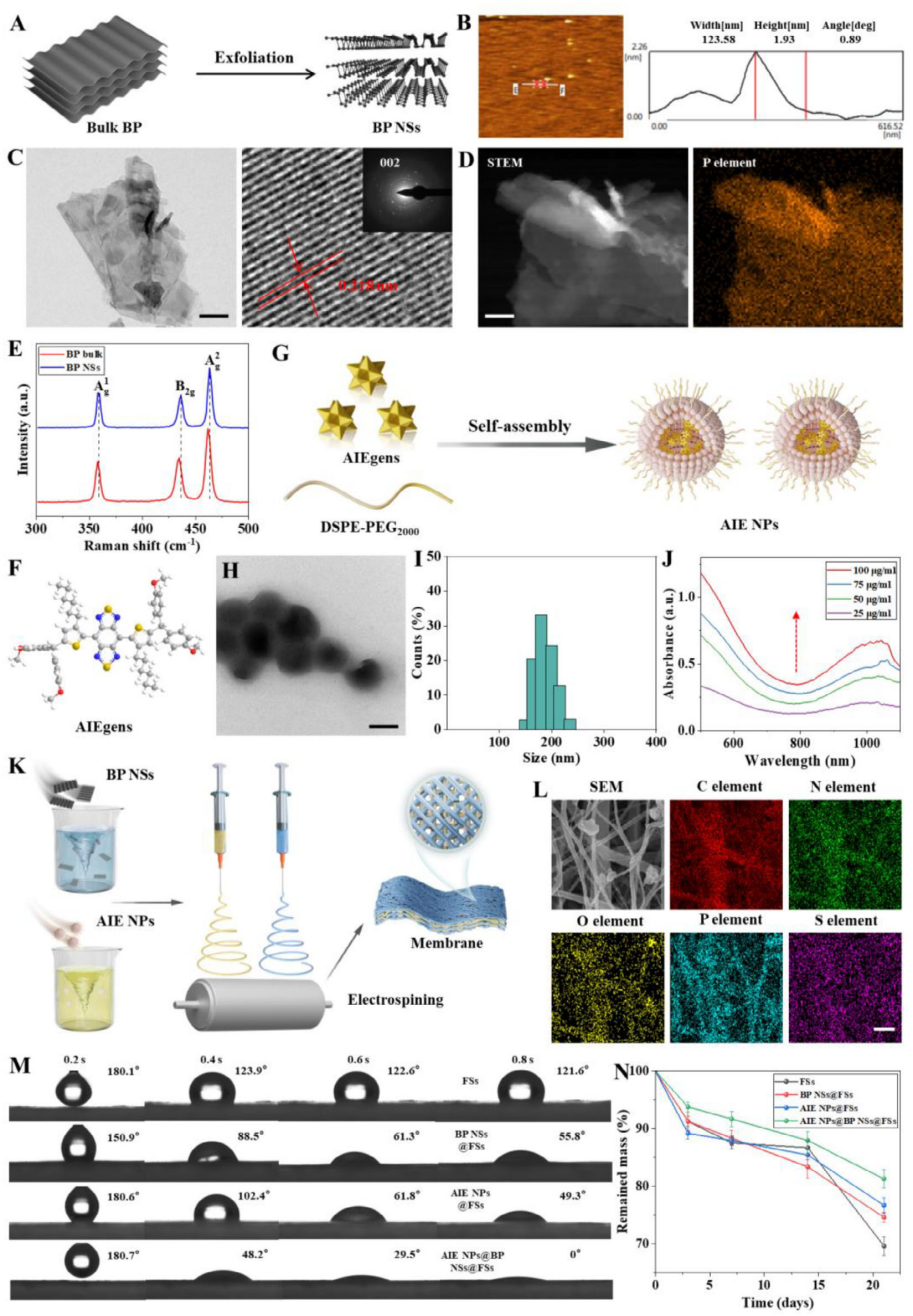
Synthesis and Multimodal Characterization of AIE NPs@BP NSs@FSs Composite System. A) Structural configuration of BP NSs preparation. B) AFM topographic profile of BP NSs. C) TEM image (left) and HR‐TEM image (right) of BP NSs, Scale bar, 10 nm. D) SEM image and elemental mapping analysis of BP NSs. Scale bar, 10 nm. E) Raman spectra of BP bulk and BP NSs. F) Schematic of AIEgen structure. G) Schematic of AIE NPs preparation. H) TEM visualization of AIE NPs. Scale bar, 200 nm. I) Hydrodynamic size distribution of AIE NPs. J) UV spectra of AIE NPs. K) Schematic representation of AIE NPs@BP NSs@FSs prepared by electrospinning. L) Elemental mapping of AIE NPs@BP NSs@FSs. Scale bar, 1 µm. M) Comparative hydrophilicity assessment via water contact angle measurements. N) Degradation curves of the four fiber membranes at 0, 3, 7, 14, and 21 d (data presented as mean ± s.d., N = 3).

The 2D BP NSs were synthesized using ultrasound‐assisted liquid exfoliation of BP bulks (**Figure** **2**A).^[^ ^35^ ^]^ The layered architecture and surface topography of BP NSs were analyzed using atomic force microscopy (AFM), which revealed that the flakes consisted of different layers with thicknesses ranging from 0.23 to 1.93 nm (Figure 2B). Transmission electron microscopy (TEM) revealed that the exfoliated BP NSs exhibited a layered morphology, with lateral dimensions ranging from 60 to 120 nm. The clear lattice fringes observed in high‐resolution TEM, which show a d‐spacing of 0.218 nm, are assignable to the (002) plane of crystalline BP (Figure 2C). Scanning electron microscopy (SEM) analysis further confirmed the 2D nanosheet structure of BP NSs (Figure 2D). The corresponding elemental mapping and energy dispersive spectroscopy (EDX) analysis of BP NSs confirmed the existence of P elements in the nanosheets (Figure 2D; Figure S1, Supporting Information). Raman spectral analysis confirmed the presence of three characteristic peaks of BP NSs, which exhibited a slight red shift relative to bulk BP, attributed to decreased layer thickness, thereby substantiating the successful exfoliation of BP NSs (Figure 2E).^[^ ^36^ ^]^

Then, AIEgens were encapsulated with DSPE‐PEG_2000_ using a simple one‐step method based on the previous study to form AIE NPs (Figure 2G).^[^ ^37^ ^]^ A schematic of the AIEgen structure is given in Figure 2F. As revealed by TEM and dynamic light scattering (DLS), the AIE NPs were monodisperse spherical particles with a mean size of ≈234 nm (Figure 2H,I). Additionally, the NPs exhibited a negative surface charge, evidenced by a zeta potential measurement of −24.02 mV (Figure S2, Supporting Information). The lowest unoccupied molecular orbital (LUMO) was primarily concentrated on the benzo[1,2‐c:4,5‐c']bis([1,2,5]thiadiazole) (BBTD) core, whereas the highest occupied molecular orbital (HOMO) was distributed across the entire conjugated backbone of the AIEgen, as illustrated in Figure S3 (Supporting Information). With a calculated HOMO‐LUMO energy gap of 1.75 eV, the material exhibited strong absorption in the NIR‐II region. The UV–vis spectra of AIE NPs exhibited broad and intense absorption bands in this region, which intensified with increasing nanoparticle concentration (Figure 2J). Finally, AIE NPs@BP NSs@FSs were prepared by incorporating AIE NPs and BP NSs into the electrostatically spun fibers (Figure 2K), and polycaprolactone (PCL) fibrous membranes were also prepared as the control group. AIE NPs and BP NSs were visibly included in the fiber composition (Figure S4, Supporting Information). The average diameter of four types of fibers ranged from ≈0.3 to 0.4 µm (Figure S5, Supporting Information). The elementary composition analyses of different membranes identified that C, N, O, S, and P were observed in AIE NPs@BP NSs@FSs membranes, which confirmed their successful formation (Figure 2L; Figure S6, Supporting Information). Water contact angle analysis showed that AIE NPs@BP NSs@FSs exhibited rapid wettability, with the angle dropping from 122.6° to 29.5° at 0.6 s and complete absorption by 0.8 s (Figure 2M), indicating markedly enhanced hydrophilicity due to AIE NPs and BP NSs doping. Tensile stress‐strain measurements revealed that the incorporation of BP NSs and AIE NPs enhanced the tensile strength (Figure S7, Supporting Information).

Biodegradability was a prerequisite for bone repair biomaterial application. Therefore, the degradation properties of four fiber membranes in phosphate‐buffered saline (PBS) were demonstrated by SEM and weight analysis. As shown in the mass loss curve graph, all groups experienced rapid mass loss in the first week, followed by a gradual decline (Figure 2N). The weight loss of AIE NPs@BP NSs@FSs reached nearly 20% after 21 d. SEM images also confirmed the above degradation behaviors of different fiber membranes after 21 d (Figure S8, Supporting Information). The Fourier transform infrared spectra (FTIR) revealed relevant characteristic peaks appeared in four fiber membranes (Figure S9A, Supporting Information), AIE NPs@BP NSs@FSs exhibited telescopic vibrational peaks at 1100–1300 cm^−1^ for P = O and 1000–1400 cm^−1^ for S = O, and all of them decreased gradually with the time of the fiber membranes immersed in PBS solution (Figure S10A–D, Supporting Information). The mineral content was assessed by thermogravimetric analysis (TGA), showing <5% weight loss at 300 °C for all membranes, indicating excellent thermal stability (Figure S9B, Supporting Information). Upon degradation, BP NSs underwent irreversible oxidation and hydrolysis, generating intermediate phosphorus oxides (P_x_O_y_) that subsequently transformed into PO_4_^3−^, leading to a progressive increase in elemental phosphorus concentration in simulated body fluid (SBF).^[^ ^38^, ^39^ ^]^ The fiber membranes were continuously immersed in SBF solution for 21 d, and the cumulative elemental P release concentration in the SBF solution was determined after 3, 7, 14, and 21 d, respectively (Figure S11, Supporting Information). To unequivocally identify the source of released phosphate, the AIE NPs@BP NSs@FSs were subjected to X‐ray photoelectron spectroscopy (XPS) analysis after being degraded in SBF for 7 days (Figure S12, Supporting Information), indicating PO_4_^3−^ derived from the oxidation of BP NSs, remained in the scaffold during degradation, thereby supporting the observed sustained phosphate release behavior. The released elemental P concentration from AIE NPs@BP NSs@FSs increased progressively, indicating the sustained degradation of BP NSs, which would contribute to enhancing remineralization and facilitating osteogenesis.^[^ ^40^, ^41^, ^42^ ^]^

### 2.2 Photothermal and ROS Scavenging Performances of AIE NPs@BP NSs@FSs

**Figure 3:**
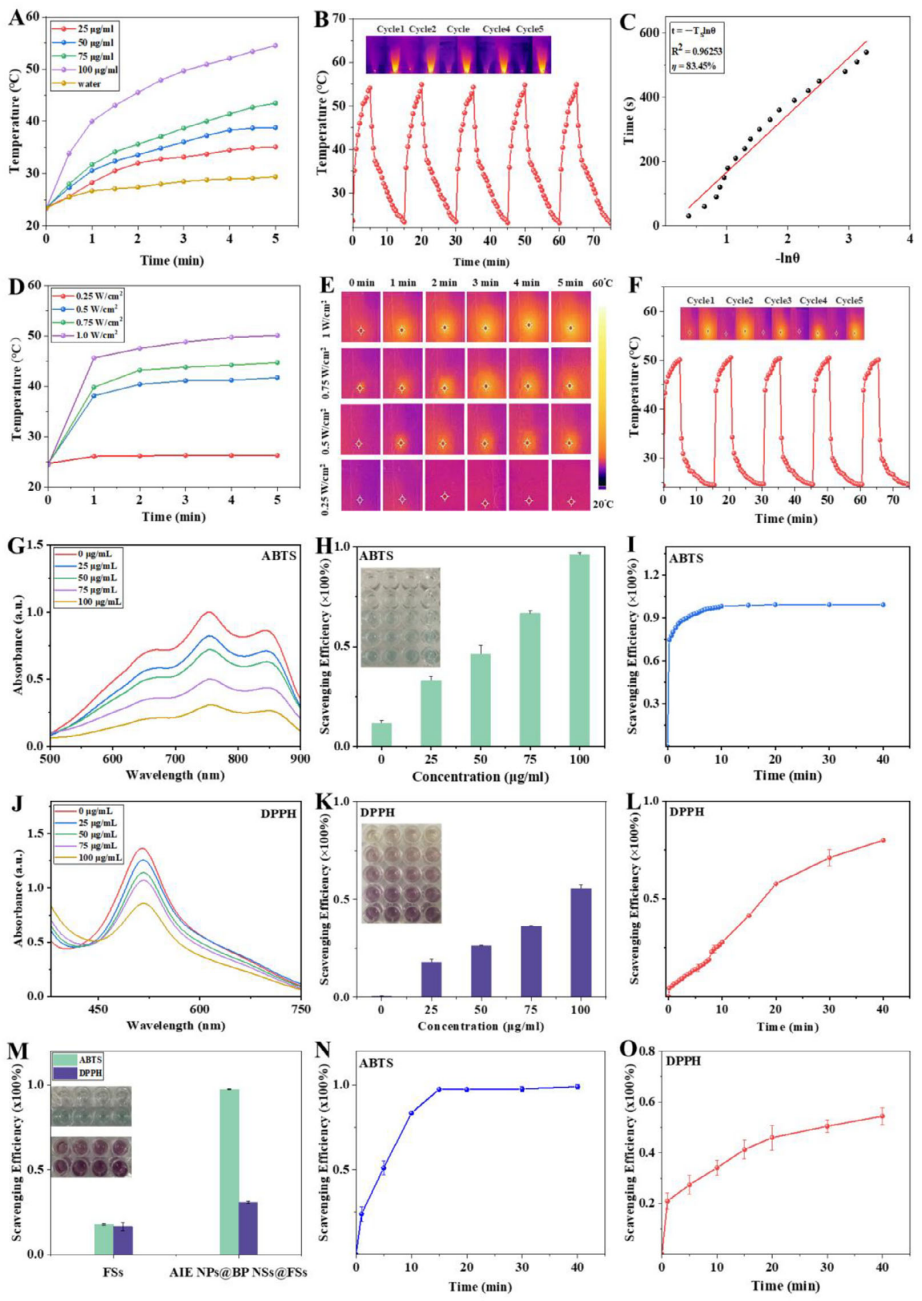
Photothermal and ROS scavenging performances of AIE NPs@BP NSs@FSs. A) Temperature change curves of different concentrations of AIE NPs under 1064 nm laser irradiation (1 W cm^2^, 5 min). B) Cyclic photothermal stability of AIE NPs across five laser ON/OFF phases. C) Photothermal conversion efficiency of AIE NPs. D) Temperature change curves and E) infrared thermograms of AIE NPs@BP NSs@FSs under a 1064 nm laser irradiation at different power densities. F) Stability of AIE NPs@BP NSs@FSs across repeated irradiation cycles. G) The absorption peak of ABTS·^+^ was treated with different concentrations of BP NSs. H) Free radical scavenging rate of ABTS·^+^ treated with different concentrations of BP NSs within 10 min. I) Free radical scavenging of ABTS·^+^ treated with 100 µg mL^−1^ of BP NSs. J) The absorption peak of DPPH· was treated with different concentrations of BP NSs. K) Scavenging rate of DPPH· treated with different concentrations of BP NSs within 20 min. L) Free radical scavenging kinetics of DPPH· treated with 100 µg mL^−1^ BP NSs. M) Free radical scavenging rates of ABTS·^+^ and DPPH· after different treatments at 20 min. N) Free radical scavenging kinetics of ABTS·^+^ treated with AIE NPs@BP NSs@FSs. O) Free radical scavenging kinetics of DPPH· treated with AIE NPs@BP NSs@FSs (data presented as mean ± s.d., N = 3).

To investigate the potential of AIE NPs@BP NSs@FSs as effective NIR‐II PTAs for deep tissue applications, the AIE NPs were irradiated with a 1064 nm laser, and their performance was characterized. The findings indicated that the photothermal performance of AIE NPs was dependent on both concentration and laser power density. Under 1064 nm laser irradiation (1.0 W cm^2^), the AIE NPs suspension (100 µg/mL) exhibited a pronounced photothermal effect, with its temperature rising by 31 °C (from 23 to 54 °C) within 5 min (**Figure** **3**A; Figures S13 and S14, Supporting Information). We further investigated the photothermal stability of AIE NPs. The results demonstrated that AIE NPs solutions maintained a consistent level of temperature after five heating‐cooling cycles under a 1064 nm laser irradiation (Figure 3B). Calculation of the photothermal conversion efficiency following a reported method yielded a value of 83% for the AIE NPs (Figure 3C), which is comparable to the performance of leading PTAs.^[^ ^43^ ^]^ The tolerance temperature of the oral mucosa is usually ≈55–60 °C. Therefore, there is no risk of these doses and irradiation intensities (100 µg mL^−1^, 1 W cm^2^) causing mucosal burns.^[^ ^44^ ^]^ Based on these findings, the combination of 100 µg mL^−1^ and 1.0 W cm^2^ was employed for all ensuing experimental work.

A systematic evaluation was conducted to study the photothermal behavior of BP NSs under 1064 nm laser irradiation using different concentrations and power densities. The temperature of the BP NSs solution rose gradually in response to higher concentrations and greater laser power, attaining a maximum of ≈30 °C following 5 min of 1064 nm irradiation (Figures S15 and S16, Supporting Information). This result aligns with prior studies demonstrating that BP's robust photothermal effects are largely confined to the NIR‐I region, with significantly weaker responses under NIR‐II excitation, further validating the necessity of incorporating NIR‐II‐responsive AIEgens for effective PTT in our design.^[^ ^26^, ^27^, ^28^, ^29^ ^]^ To further investigate the photothermal performance of AIE NPs@BP NSs@FSs, the membrane was placed in 24‐well plates, and the photothermal performance (Figure 3D,E) and photothermal stability (Figure 3F) were assessed. The photothermal response of AIE NPs@BP NSs@FSs demonstrated a clear power‐dependent behavior, with the temperature rapidly rising to 50 °C within 5 min (1064 nm, 1 W cm^2^). The system also exhibited excellent thermal stability across five repeated heating‐cooling cycles. These findings highlighted excellent photothermal conversion performance and robust stability of AIE NPs@BP NSs@FSs.

The ROS scavenging activities of BP NSs and AIE NPs@BP NSs@FSs were assessed using the standard ABTS·^+^ and DPPH· radical assays. The unique physical structure of BP NSs resulted in BP NSs effectively acting as an antioxidant for scavenging ROS.^[^ ^45^, ^46^ ^]^ We measured the absorbance at 734 nm; the peak intensity of ABTS·^+^ decreased gradually with increasing concentrations of BP NSs (Figure 3G). Furthermore, the ABTS·^+^ radical scavenging rate attained 95% within 10 min at a concentration of 100 µg/mL (Figure 3H), and almost all of the ABTS·^+^ radicals can be scavenged within 20 min (Figure 3I). With increasing concentrations of BP NSs, the characteristic absorbance peak of the DPPH· radical at 517 nm exhibited a gradual diminishment in intensity (Figure 3J). The DPPH· radical scavenging rate rose as the concentration of BP NSs increased within 20 min (Figure 3K). After treatment with 100 µg mL^−1^ BP NSs, 55% of DPPH· radicals were eliminated within 20 min (Figure 3L). The final free radical scavenging ability of AIE NPs@BP NSs@FSs was further verified by ABTS·^+^ and DPPH· radical assay (Figure 3M). AIE NPs@BP NSs@FSs showed significantly higher scavenging efficiency of ABTS·^+^ radicals, reaching almost 100% within 20 min (Figure 3N), and higher scavenging of DPPH· radicals, reaching almost 55% within 40 min (Figure 3O). To further assess the stability of this ROS‐scavenging performance under therapeutic conditions, we evaluated the radical‐scavenging capacity of AIE NPs@BP NSs@FSs under NIR‐II laser irradiation (1.0 W cm^2^, 10 min) (Figure S17A, Supporting Information). The results showed that the ABTS·⁺ and DPPH·scavenging kinetics remained nearly unchanged compared to the non‐irradiated group, achieving nearly 97% ABTS·⁺ radical scavenging within 20 min (Figure S17B, Supporting Information) and ≈50% DPPH· radical scavenging within 40 min (Figure S17C, Supporting Information). These values were comparable to those observed under non‐irradiated conditions, indicating that AIE NPs@BP NSs@FSs maintain stable and efficient ROS scavenging capability even upon NIR‐II laser exposure. Additionally, Electron Spin Resonance (ESR) spectroscopy demonstrated that AIE NPs@BP NSs@FSs effectively scavenged representative ROS species, including •O_2_^−^, •OH, and ONOO^−^ (Figure S18A–C, Supporting Information), confirming its broad‐spectrum antioxidative capability.

### 2.3 Biocompatibility and Antimicrobial Properties of AIE NPs@BP NSs@FSs

**Figure 4:**
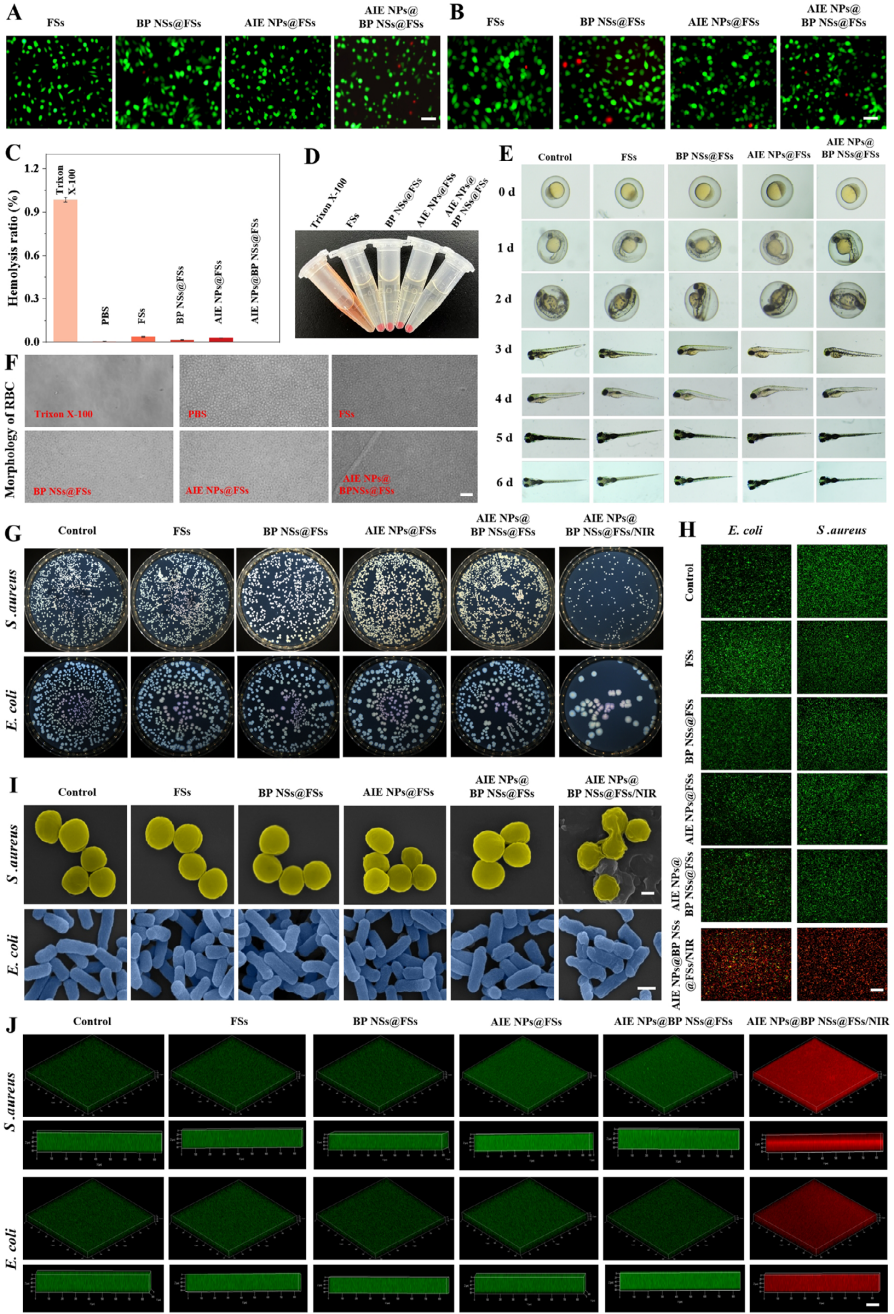
Biocompatibility and antimicrobial properties of AIE NPs@BP NSs@FSs. A) Live/dead staining of L929 fibroblasts across experimental groups. Scale bar, 100 µm. B) Parallel viability analysis in HOK cells across experimental groups. Scale bar, 100 µm. C) Quantitative hemolytic potential of erythrocytes. D) Macroscopic hemolysis visualization. E) Developmental outcome of zebrafish after different treatments. F) Morphology of erythrocytes after different treatments under the microscope. Scale bar, 100 µm. G) *E. coli* and *S. aureus* colonies after different treatments. H) Bacterial viability profiling via SYTO 9/PI dual staining. Scale bar, 100 µm. I) Ultrastructural deformation of pathogens by SEM. Scale bar, 1 µm. J) Live/dead staining of biofilms formed under therapeutic intervention. Scale bar, 100 µm (data presented as mean ± s.d., N = 3).

The in vitro cytocompatibility of the AIE NPs@BP NSs@FSs was evaluated with L929 fibroblasts and human oral keratinocytes (HOK) using Cell Counting Kit‐8 (CCK‐8) assays and live/dead staining. No significant difference in cell survival was observed between the AIE NPs@BP NSs@FSs‐treated group and the control (Figure S19, Supporting Information). The live/dead fluorescent staining further confirmed that most cells exhibited green fluorescence, indicating high cell viability in any of the four electrostatically spun membranes after 24 h of co‐culture (**Figure** **4**A,B). The hemocompatibility of AIE NPs@BP NSs@FSs was analyzed by hemolysis analysis (Figure 4C,D). Compared with the complete hemolysis induced by Triton X‐100, there was no significant change in erythrocyte morphology after co‐culturing with four membranes for 1 h, suggesting that AIE NPs@BP NSs@FSs had good hemocompatibility (Figure 4F). AIE NPs@BP NSs@FSs were co‐cultured with zebrafish embryos. After 6 d of observation, the embryos displayed normal growth and differentiation, with no evident developmental abnormalities (Figure 4E).

Prompted by its excellent photothermal performance, the NIR‐II‐induced antibacterial activity of AIE NPs@BP NSs@FSs was assessed in vitro using models of *S. aureus* and *E. coli*. Following the application of 1064 nm laser light at a power density of 1.0 W cm^2^ for 10 min, colony counts showed a significant decrease in CFUs compared to controls, confirming effective photothermal bacterial killing (Figure 4H). Live/dead staining further showed mostly red fluorescence (PI) in the treated group, indicating bacterial membrane damage, while the control groups mainly showed green (SYTO9) staining. SEM images supported these results, revealing severe membrane damage in bacteria after treatment, whereas controls maintained intact morphology (Figure 4I). Given that biofilms function as protective barriers for bacteria in wound infections, the efficacy of AIE NPs@BP NSs@FSs in disrupting these biofilms was also evaluated.^[^ ^47^, ^48^ ^]^ Live/dead staining displayed strong green fluorescence in control and untreated groups (Figure 4J). Conversely, AIE NPs@BP NSs@FSs with NIR irradiation caused bright red fluorescence, suggesting effective biofilm disruption through photothermal bacterial killing. Flow cytometry further confirmed decreased bacterial viability and abnormal distribution following treatment (Figure S20, Supporting Information). Crystal violet staining showed a marked reduction in biofilm biomass in the AIE NPs@BP NSs@FSs/NIR group (Figure S21, Supporting Information), along with impaired biofilm formation (Figure S22, Supporting Information). Additionally, qPCR analysis revealed increased expression of key anti‐biofilm genes (*sspA*, *arcC*, *arcD*, and *argF*) (Figure S23, Supporting Information), indicating a transcriptional mechanism behind biofilm suppression. Overall, AIE NPs@BP NSs@FSs successfully eliminate planktonic bacteria and disrupt biofilms via photothermal effects.

### 2.4 Immunomodulatory Properties of AIE NPs@BP NSs@FSs

**Figure 5:**
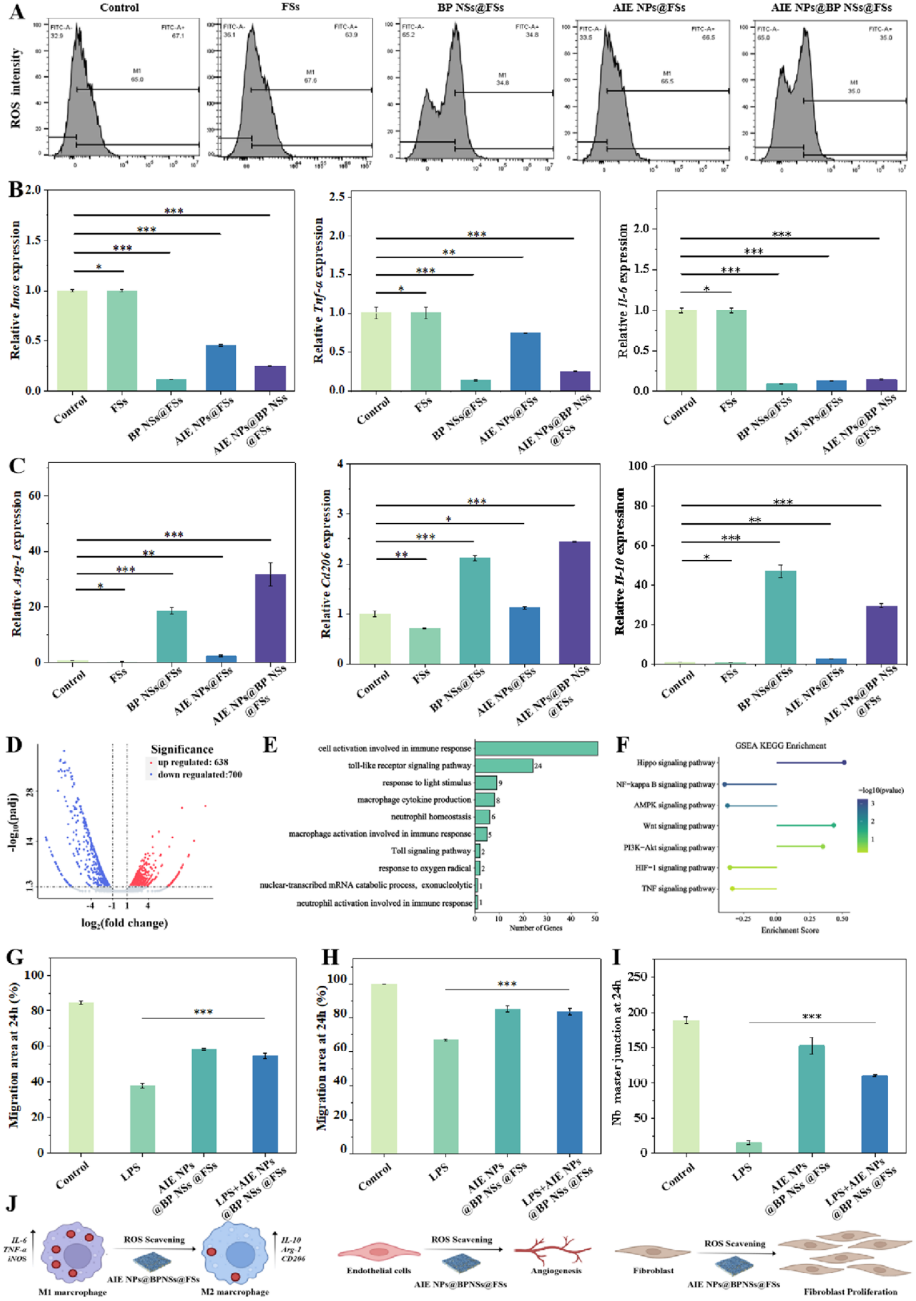
Immunomodulatory properties of AIE NPs@BP NSs@FSs. A) Flow cytometry analyses of RAW264.7 cells under different treatments. B) Transcriptional profiling of M1‐associated markers (*Inos*, *Tnf‐α*, and *IL‐6*) under differential therapeutic interventions. C) Expression of RAW264.7 anti‐inflammatory‐related genes (*Arg‐1*, *Cd206*, and *Il‐10*) cultured under different treatments. D) Volcano plot of the difference in the expression of RAW264.7 in the AIE NPs@BP NSs@FSs‐treated group versus control. E) GO enrichment analysis of DEG between AIE NPs@BP NSs@FSs‐treated group versus GO enrichment analysis of DEG between the control group. F) KEGG enrichment analysis of DEG between AIE NPs@BP NSs@FSs‐treated and control group. G) Migration rate quantification in the MAEC cell line under different treatments at 24 h. H) Migration rate quantification in L929 cells under different treatments at 24 h. I) Quantitative analysis of endothelial tubular network formation of MAECs under LPS and different treatments at 24 h. J) Schematic illustration of the proposed mechanism by which AIE NPs@BP NSs@FSs promote tissue regeneration within the inflammatory microenvironment: AIE NPs@BP NSs@FSs mediate ROS scavenging, promote M2 macrophage polarization, enhance fibroblast proliferation, and stimulate endothelial angiogenesis. Data are shown as mean ± s.d. (*p < 0.05, **p < 0.01, ***p < 0.001, N = 3).

Macrophage behavior following biomaterial implantation plays a crucial role in bone regeneration.^[^ ^49^ ^]^ M2 macrophages promote osteogenesis by secreting anti‐inflammatory cytokines that enhance osteoblast differentiation.^[^ ^50^, ^51^, ^52^ ^]^ In contrast, prolonged M1 activation exacerbates inflammation, impairs bone repair, and contributes to tissue damage. Repolarization of macrophages to the M2 phenotype is therefore indispensable for effective inflammatory suppression and subsequent bone regeneration.^[^ ^46^, ^52^ ^]^ This study assessed the ROS scavenging capability of AIE NPs@BP NSs@FSs in cells using a ROS staining assay. Following LPS‐induced inflammatory polarization of RAW264.7 macrophages, therapeutic intervention with BP NSs@FSs and AIE NPs@BP NSs@FSs was implemented; fluorescence imaging revealed a marked reduction in intracellular ROS levels (Figure S24A, Supporting Information). A significant drop in ROS levels, measured by fluorescence intensity via flow cytometry, was observed in macrophages incubated with BP NSs@FSs and AIE NPs@BP NSs@FSs (**Figure** **5**A). Furthermore, the macrophage population exhibited a phenotypic transition from the M1 to the M2 subtype.^[^ ^48^, ^53^ ^]^

To further explore the regulatory effects of AIE NPs@BP NSs@FSs on macrophage polarization, this study conducted qPCR analysis. Differential gene expression analysis revealed that M1 markers (*Inos, Tnf‐α, Il‐6*) were downregulated, while M2 markers (*Arg‐1, Cd206, Il‐10*) were upregulated in cells treated with BP NSs@FSs or AIE NPs@BP NSs@FSs (Figure 5B,C). To further investigate the potential anti‐inflammatory mechanisms by which AIE NPs@BP NSs@FSs affected macrophage phenotype, this study performed RNA sequencing (RNA‐seq) analysis. RNA‐seq revealed 1338 differentially expressed genes (DEGs) between the AIE NPs@BP NSs@FSs‐treated group and the untreated control, with 638 upregulated and 700 downregulated (Figure 5D). Transcriptional variants underwent systematic functional annotation through Gene Ontology (GO) and Kyoto Encyclopedia of Genes and Genomes (KEGG) pathway mapping. GO classification delineated these DEGs into three functional domains: biological processes (BP), cellular components (CC), and molecular functions (MF) (Figure 5E). KEGG enrichment analysis revealed that DEGs were significantly enriched in inflammation‐related signaling pathways (Figure 5F).

Notably, a more refined transcriptomic analysis indicated a clear divergence in pathway enrichment patterns: downregulated genes were predominantly associated with pro‐inflammatory pathways, including NF‐κB and TNF signaling, suggesting suppression of inflammatory responses. In contrast, upregulated genes were significantly involved in immunoregulatory and anti‐inflammatory pathways, reflecting a shift toward M2‐like macrophage polarization. In the progression of infectious inflammation, LPS binds to Toll‐like receptor 4 (TLR4), which leads to the phosphorylation and degradation of IκBα. This subsequently promotes the nuclear translocation of NF‐κB p65, activating the expression of downstream inflammatory cytokines, including TNF‐α, iNOS, and IL‐6 (Figure S25A, Supporting Information).^[^ ^54^ ^]^ To delve deeper into the mechanistic basis, we assessed the activity of the NF‐κB signaling pathway in RAW264.7 cells following a 24 h treatment period with various treatments. Western blot analysis showed that the LPS group exhibited increased phosphorylation of IκBα and p65, along with decreased total IκBα levels due to degradation. In contrast, AIE NPs@BP NSs@FSs treatment reversed these phosphorylation changes without altering total IκBα or p65 expression, indicating inhibition of NF‐κB pathway activation (Figure S25B, Supporting Information). Quantitative analysis further confirmed this effect (Figure S26A,B, Supporting Information). These findings further demonstrate that AIE NPs@BP NSs@FSs negatively regulate NF‐κB signaling and contribute to their anti‐inflammatory effects.

To further investigate the mechanisms by which AIE NPs@BP NSs@FSs mediated immunomodulation, L929 and mouse aortic endothelial cells (MAECs) were treated with LPS, and then the impact of AIE NPs@BP NSs@FSs on cell proliferation and migration was observed.^[^ ^55^, ^56^ ^]^ The results showed that LPS treatment significantly inhibited MAECs proliferation, whereas AIE NPs@BP NSs@FSs treatment effectively mitigated this inhibitory effect (Figure S24B,C, Supporting Information). Similarly, LPS treatment markedly impaired the migratory capacity of MAECs; however, AIE NPs@BP NSs@FSs treatment reversed this effect, restoring cell motility (Figure 5G; Figures S27 and S28, Supporting Information). Furthermore, the results indicated that LPS treatment moderately inhibited both the proliferation and migration of L929 fibroblast cells, while the AIE NPs@BP NSs@FSs treatment reversed this effect (Figure 5H; Figures S29–S31, Supporting Information). Then, the effects of AIE NPs@BP NSs@FSs on angiogenesis were further evaluated using a Matrigel tube formation assay.^[^ ^57^ ^]^ Morphometric evaluation demonstrated significant attenuation of vascular network density in LPS‐challenged models compared to controls, linking inflammatory milieu‐mediated suppression of neovascularization. However, a substantial formation of primary vasculature‐like networks was observed after treatment with AIE NPs@BP NSs@FSs (Figure S32, Supporting Information). In addition, both the LPS+AIE NPs@BP NSs@FSs‐treated group and the AIE NPs@BP NSs@FSs‐treated group were significantly higher than the LPS‐treated group in terms of the total number of connections at 6 and 24 h (Figure 5I; Figure S33, Supporting Information). Overall, these results demonstrate that AIE NPs@BP NSs@FSs can effectively scavenge ROS within the inflammatory microenvironment, driving macrophage immunophenotypic reprogramming from M1 to M2, enhancing L929 fibroblast proliferation, and stimulating MAEC‐mediated angiogenesis. The proposed mechanism is illustrated in the schematic diagram (Figure 5J), highlighting the immunomodulatory and pro‐regenerative functions of AIE NPs@BP NSs@FSs.

### 2.5 in vitro Bone Immunomodulation by AIE NPs@BP NSs@FSs

**Figure 6:**
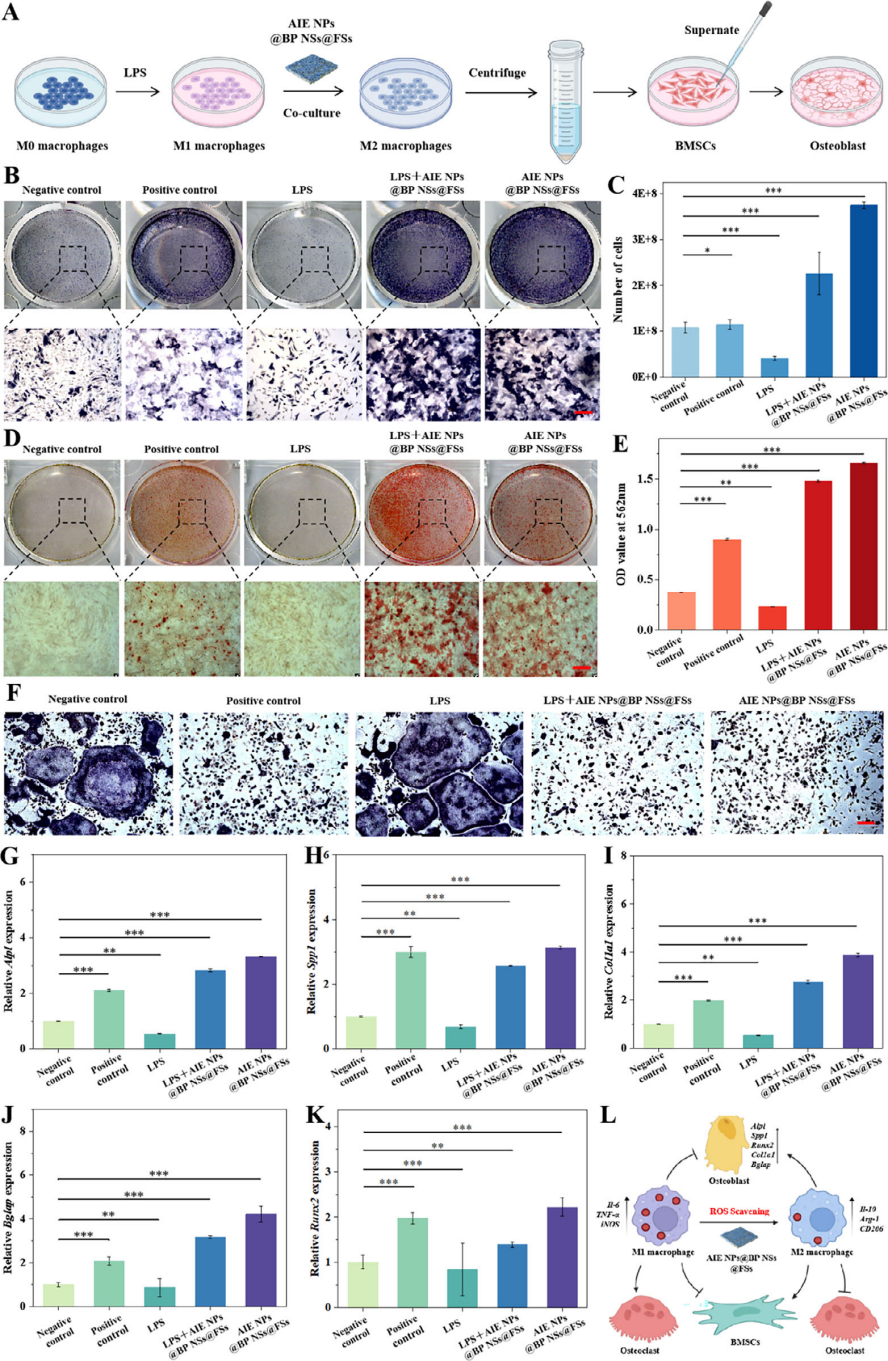
In vitro bone immunomodulation of AIE NPs@BP NSs@FSs. A) Schematic diagram of the osteogenic differentiation mechanism of BMSCs cultured with AIE NPs@BP NSs@FSs under simulated inflammatory conditions. B) Multiscale visualization (macro/micro) of ALP enzymatic activity in BMSC‐derived osteoblasts across experimental cohorts. Scale bar, 100 µm. C) Semi‐quantitative ALP enzymatic activity profiling. D) Multiscale visualization (macro/micro) of ARS staining in BMSC‐derived osteoblasts across experimental cohorts. Scale bar, 100 µm. E) ARS calcium nodule content analysis. F) TRAP+ osteoclastogenesis in RAW 264.7 cells post‐intervention. Scale bar, 200 µm. qPCR analysis of osteogenic marker gene expression on day 7, including G) *Alpl*, H) *Spp1*, I) *Col1a1*, J) *Bglap*, and K) *Runx2*. L) Schematic representation of AIE NPs@BP NSs@FSs promoting osteoblast differentiation and inhibiting osteoclast formation by M2 polarization. Data are shown as mean ± s.d. (*p < 0.05, **p < 0.01, ***p < 0.001, N = 3).

Osteoimmunology research has demonstrated a close link between immune‐inflammatory responses and new bone formation.^[^ ^58^, ^59^, ^60^ ^]^ To verify this relationship, we assessed how macrophage‐secreted cytokines under various treatments influenced primary rat bone marrow mesenchymal stem cells (BMSCs). As depicted in **Figure** **6**A, an inflammatory model was initially generated by exposing RAW264.7 cells to LPS, then RAW264.7 cells were treated with AIE NPs@BP NSs@FSs, cytokine‐containing supernatants were collected from both the control and experimental groups, and subsequently used to stimulate BMSCs. The progression of osteogenesis was assessed through histochemical quantification of alkaline phosphatase (ALP) activity (early differentiation marker) and calcium deposition via Alizarin Red S (ARS) staining (late mineralization phase). BMSCs were incubated under various conditions for 14 d. The negative control group received no osteogenic‐inducing factors, while the positive control group was supplemented with osteogenic‐inductive factors. Additionally, BMSCs were directly cultured with AIE NPs@BP NSs@FSs, and ALP staining was performed across all groups to evaluate osteogenic activity (Figure 6B). The LPS‐treated group showed the lowest staining intensity, while the AIE NPs@BP NSs@FSs‐treated group exhibited the most intense ALP staining. Similarly, the LPS+AIE NPs@BP NSs@FSs‐treated group also demonstrated osteogenesis‐promoting effects. Semiquantitative ALP analysis further confirmed these findings (Figure 6C). The result demonstrated suppressed ALP biosynthesis in LPS‐stimulated macrophages compared to control, while the AIE NPs@BP NSs@FSs therapeutic intervention counteracted inflammatory suppression. ARS quantification confirming enhanced calcium nodule formation (Figure 6D), where a large amount of crimson calcium accumulation was observed in BMSCs in the AIE NPs@BP NSs@FSs‐treated group and LPS+AIE NPs@BP NSs@FSs‐treated group after 21 d of treatment, corroborating the histomorphometric quantification of calcium nodules, the AIE NPs@BP NSs@FSs‐treated group exhibited an absorbance at 562 nm approximately twofold higher than the control, whereas the LPS group showed the lowest value (Figure 6E). The LPS‐treated group displayed markedly attenuated osteogenic capacity, consistent with inflammatory milieu‐driven macrophage polarization toward the M1 phenotype and subsequent pro‐osteoclastogenic cytokine cascade. AIE NPs@BP NSs@FSs counteracted inflammatory osteogenic suppression by driving macrophage functional plasticity toward M2 immunoregulatory polarization to potentiate bone matrix deposition.

Studies have shown that an inflammatory microenvironment enhances osteoclast differentiation and accelerates bone resorption.^[^ ^61^ ^]^ In this study, osteoclastogenesis was induced by treating RAW264.7 cells with various conditions (negative control, positive control, LPS, LPS + AIE NPs@BP NSs@FSs, AIE NPs@BP NSs@FSs), with RANKL and M‐CSF administration standardized across all groups except the control group. TRAP+ osteoclastogenesis was histomorphometrically assessed using tartrate‐resistant acid phosphatase (TRAP) cytochemical detection across treatment groups (Figure 6F). The negative control and LPS‐treated groups exhibited similar results, with a high count of multinucleated giant cells. A significant reduction in osteoclast count was observed in the LPS plus AIE NPs@BP NSs@FSs treatment group. In the AIE NPs@BP NSs@FSs‐treated group, osteoclasts were rarely detected. To further evaluate osteogenic differentiation, osteogenesis‐related genes were screened, and their expression levels were quantified by qPCR (Figure 6G–K). After 7 days of osteogenic induction, the greatest level of transcriptional activation for key osteogenic markers (*Alpl, Spp1, Col1a1, Bglap*, and *Runx2*) was detected in the AIE NPs@BP NSs@FSs group, in contrast to the LPS group, which showed the weakest expression.

Skeletal homeostasis is governed by osteoclast‐osteoblast coupling.^[^ ^60^, ^61^ ^]^ As demonstrated in previous experiments, AIE NPs@BP NSs@FSs orchestrated macrophages from M1 proinflammatory dominance to M2 immunoregulatory polarization through ROS scavenging. M1 macrophages are known to secrete pro‐inflammatory cytokines that inhibit osteogenesis. Conversely, M2 macrophages release anti‐inflammatory cytokines that stimulate osteogenic differentiation while simultaneously inhibiting osteoclast activity, thereby upregulating osteogenic gene expression (*Alpl, Spp1, Col1a1, Bglap*, and *Runx2*) in BMSCs. Thus, this study elucidated the mechanism of action of AIE NPs@BP NSs@FSs in bone regeneration (Figure 6L).

### 2.6 In Vivo Infected Bone Regeneration

**Figure 7:**
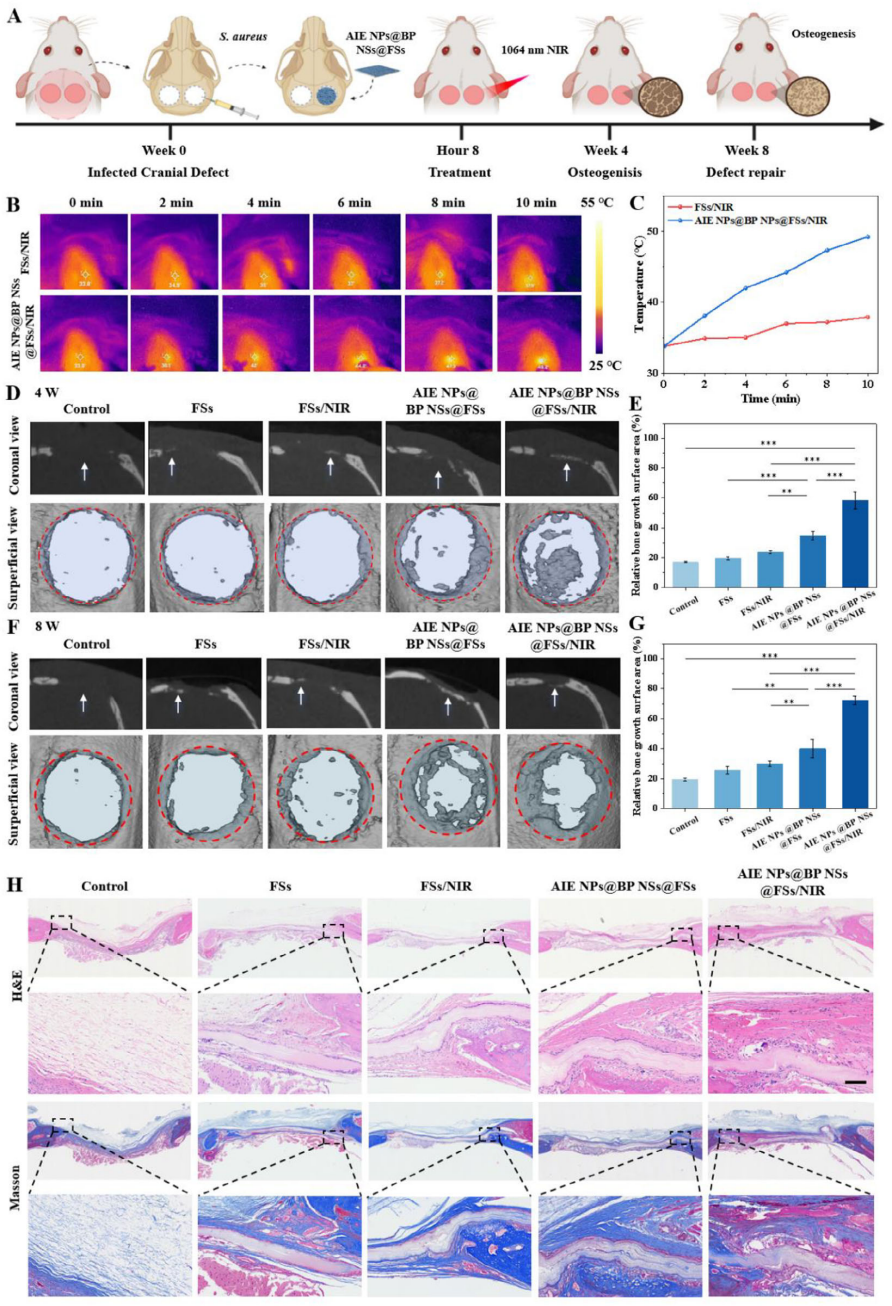
In vivo effects of AIE NPs@BP NSs@FSs/NIR in repairing infectious bone defects. A) Experimental design timeline for therapeutic monitoring. B) Infrared thermography photos and C) temperature changes of FSs‐treated and AIE NPs@BP NSs@FSs/NIR‐treated groups. D) Coronal/sagittal micro‐CT reconstruction (4‐week postoperative) in each group. E) Quantification of relative bone growth surface area at the defect sites across different groups at week 4. F) Longitudinal micro‐CT reconstruction (8‐week follow‐up). G) Quantitative analysis of relative bone growth surface area at week 8. H) Histological evaluation at 8 weeks using H&E and Masson trichrome staining, an enlarged view of the selected area is shown (black dashed box). Scale bar, 100 µm. Data are shown as mean ± s.d. (**p < 0.01, ***p < 0.001, N = 3).

The antibacterial and osteogenic efficacy of AIE NPs@BP NSs@FSs combined with NIR irradiation was further assessed using a rat model of infected critical‐sized cranial defects. A standardized 5 mm calvarial defect was surgically created in Sprague‐Dawley (SD) rats, followed by the injection of *S. aureus* to establish a localized bone infection model. Subsequently, different materials were implanted in the defects for different treatments (**Figure** **7**A). To further confirm the in vivo biosafety of our scaffold, Hematoxylin and eosin (H&E) staining was performed on the heart, liver, spleen, lung, and kidney tissues of SD rats after implantation. The results revealed no observable pathological abnormalities compared to the control group, indicating that AIE NPs@BP NSs@FSs induced no significant organ toxicity (Figure S34, Supporting Information). Furthermore, hematological analysis showed no significant differences in major blood parameters (WBC, LYM, RBC, PLT, RDW, and MPV, etc.) across groups (Figure S35, Supporting Information), further confirming the systemic biocompatibility and low toxicity of the scaffold. The photothermal effect of AIE NPs@BP NSs@FSs was assessed by applying 1064 nm laser light (1.0 W cm^2^) within the NIR‐II window for 10 min, while an infrared thermal camera captured real‐time temperature distribution. As shown in Figure 7B,C, NIR‐II irradiation elevated the local temperature at the AIE NPs@BP NSs@FSs‐treated site to ≈49 °C, while the control group remained at ≈37 °C. The heating was precisely confined to the scaffold region, and histological analysis revealed no damage to surrounding tissues, nor any signs of thermal necrosis or impaired bone formation, confirming the thermal safety of our approach. Similar high‐temperature PTT strategies (>50 °C) have been reported to be safe in vivo when engineered scaffolds spatially restrict thermal effects.^[^ ^62^, ^63^, ^64^ ^]^ Furthermore, our photothermal stability tests demonstrated a rapid return to baseline temperature after irradiation, indicating the transient nature of the heating. Recent studies have also shown that localized and mild hyperthermia does not disrupt immune modulation;^[^ ^65^, ^66^, ^67^ ^]^ on the contrary, photothermal strategies reaching 50–52 °C for short durations can enhance local circulation and promote a pro‐regenerative immune microenvironment, thereby supporting bone healing.^[^ ^68^ ^]^

In the context of bone regeneration, the bone defect was almost empty in the blank group without scaffold implantation, with the defect area remaining largely unhealed. In contrast, groups treated with biomaterial scaffolds exhibited enhanced bone regeneration, likely attributed to the scaffolds' ability to provide a favorable structural framework that supports cell adhesion and proliferation. Notably, the BP NSs@FSs‐treated group demonstrated significantly greater bone formation compared to the control group. This superior osteogenic performance was explained by the gradual degradation of BP NSs, which led to the sustained release of PO_4_^3−^. These ions participate directly in the mineralization process, facilitating osteoblast‐mediated new bone deposition.^[^ ^20^, ^21^ ^]^ As shown in Figure 7D, the AIE NPs@BP NSs@FSs combined with the NIR irradiation group exhibited the most pronounced bone regeneration at week 4, with a relative bone growth surface area of ≈59% (Figure 7E). By week 8, the bone defects were well‐repaired by new bone tissue in the AIE NPs@BP NSs@FSs/NIR‐treated group (Figure 7F), and the bone growth surface area further increased to 72% (Figure 7G). Moreover, quantitative micro‐CT analysis revealed that the bone volume to tissue volume (BV/TV) and trabecular number (Tb.N) in the AIE NPs@BP NSs@FSs/NIR‐treated group reached 34.6% and 0.76 mm^−1^ at week 4, and increased to 48.9% and 1.0 mm^−1^ at week 8, respectively (Figures S36 and S37, Supporting Information), further confirming the osteopromotive effects of the AIE NPs@BP NSs@FSs‐based photothermal platform.

To evaluate bone regeneration, a histomorphometric analysis was conducted on the defect sites using H&E and Masson's trichrome‐stained sections (Figure 7H). In the control group, the defect area was mainly filled with fibrous connective tissue and showed tissue collapse. In contrast, all groups implanted with scaffolds showed evidence of new bone formation on the material surface, with bone tissue tending to grow horizontally—likely due to the physical support and spatial guidance provided by the scaffolds. Specifically, in the BP NSs@FSs‐treated group, moderate new bone formation and vascularization were observed at the periphery of the defect. In the AIE NPs@BP NSs@FSs/NIR‐treated group, extensive new bone tissue and vascular infiltration were evident not only at the edges but also in the central region of the defect, indicating a more robust and widespread bone regeneration response. The antibacterial effects of different treatments at the defect site were assessed using an agar plate assay (Figure S38, Supporting Information). A large number of *S. aureus* colonies were observed on agar plates in the control group, while treatment with AIE NPs@BP NSs@FSs combined with NIR irradiation resulted in a marked reduction in bacterial colonies. Consistent with the in vitro antimicrobial results, the quantitative analysis further confirmed that over 80% of the bacterial load was eliminated from the infected defect area after AIE NPs@BP NSs@FSs/NIR treatment (Figure S39, Supporting Information). Additionally, Gram‐stained histological sections of tissues adjacent to the infected bone defects showed no visible signs of infection in the AIE NPs@BP NSs@FSs/NIR‐treated group at 8 weeks post‐treatment, with no dark blue Gram‐positive bacterial clusters observed (Figure S40, Supporting Information). Besides eradicating infection, the AIE NPs@BP NSs@FSs/NIR platform significantly enhanced bone regeneration, confirming its dual role in antibacterial defense and osteogenesis.

### 2.7 In Vivo Anti‐Inflammatory and Promote Osteogenesis and Angiogenesis

**Figure 8:**
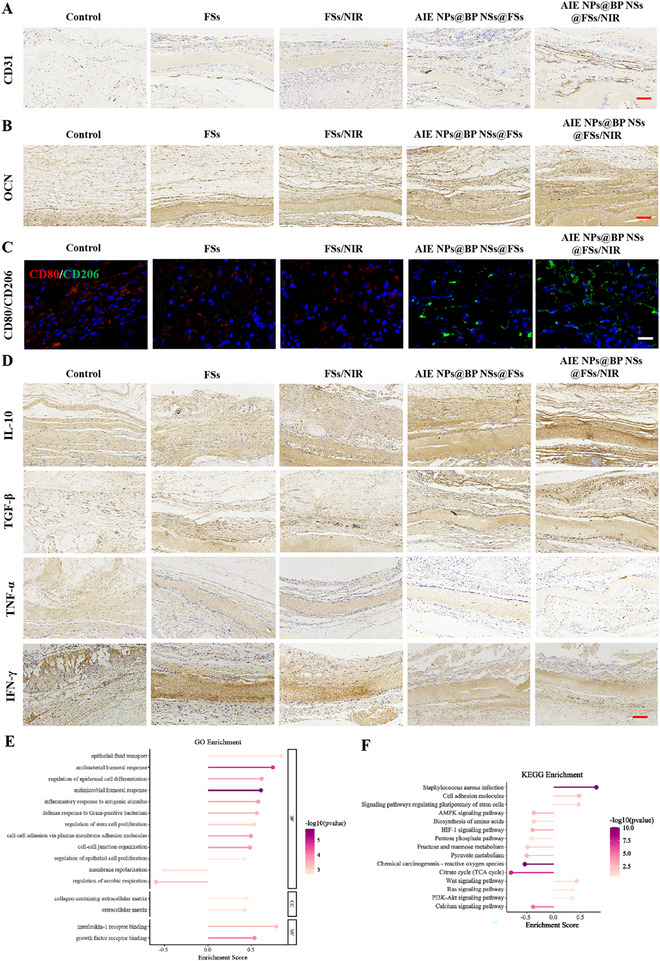
In vivo anti‐inflammatory and promotes osteogenesis and angiogenesis. A) CD31^+^ neovascularization profiles in defect zones at 8 weeks post‐treatment. Scale bar, 100 µm. B) OCN spatial distribution in defect areas at 8 weeks post‐treatment. Scale bar, 100 µm. C) Immunofluorescence co‐localization of CD206 staining (green) and CD80 staining (red) in defect areas at 8 weeks post‐treatment. Scale bar, 100 µm. D) Representative IHC images in defect areas at 8 weeks post‐treatment, including IL‐10, TGF‐β (anti‐inflammatory), and TNF‐α, IFN‐γ (pro‐inflammatory). Scale bar, 100 µm. E) GO enrichment analysis and F) KEGG pathway enrichment of differentially expressed transcripts post‐intervention.

The newly formed vasculature provides oxygen, nutrients, and signaling molecules essential for osteogenesis. In our experiments, the angiogenic potential of the biomaterials was initially assessed. CD31 was employed for immunohistochemical (IHC) staining to visualize neovascularization within the defect regions.^[^ ^47^, ^48^, ^49^ ^]^ As evidenced by **Figure** **8**A, notable CD31‐positive staining was present in groups treated with BP NSs@FSs and AIE NPs@BP NSs@FSs/NIR, which corresponded to an enhanced in vivo vascularization capability. To assess the osteogenic potential of the AIE NPs@BP NSs@FSs/NIR platform, immunohistochemical staining for osteocalcin (OCN), a late‐stage osteogenesis marker, was performed on bone sections from each group. Figure 8B illustrates pronounced OCN expression in newly formed bone areas within the BP NSs@FSs and AIE NPs@BP NSs@FSs/NIR groups, whereas only weak staining was observed in the other groups. It has been observed that the AIE NPs@BP NSs@FSs/NIR platform exhibits the greatest pro‐angiogenic and bone repair‐promoting potential in a rat cranial bone defect model (Figure S41A,B, Supporting Information). The bone regeneration process was accelerated due to the synergistic effect between newly formed blood vessels and bone.

To better understand the immunomodulatory effects of AIE NPs@BP NSs@FSs/NIR platform during the regeneration of infected bone defects, immunofluorescence staining was performed on the defect site tissues at 8 weeks post‐treatment (Figure 8C). Quantitative analysis revealed that the proportion of CD80^+^ cells in the BP NSs@FSs and the AIE NPs@BP NSs@FSs/NIR groups (9.02 ± 1.70% and 11 ± 1.06%, respectively) was significantly lower than that in the control group (44.09% ± 2.38%) (Figure S42A,B, Supporting Information), conversely, the percentages of CD206^+^ cells were markedly elevated (44.3 ± 2.85% and 54.29 ± 1.95%, respectively). IL‐10 and TGF‐β are pivotal anti‐inflammatory cytokines that drive M2 macrophage polarization and mediate immune homeostasis. In contrast, TNF‐α and IFN‐γ are major pro‐inflammatory cytokines closely associated with M1 macrophage polarization, contributing significantly to the amplification of inflammatory responses. To further validate the anti‐inflammatory efficacy of the AIE NPs@BP NSs@FSs/NIR platform, IHC profiling was systematically quantified (Figure 8D). The results showed that the AIE NPs@BP NSs@FSs/NIR‐treated group exhibited robust positive staining for IL‐10 and TGF‐β, with quantitative analysis demonstrating markedly higher expression levels relative to all other groups (Figure S43, Supporting Information). Conversely, TNF‐α and IFN‐γ expression was markedly reduced in the AIE NPs@BP NSs@FSs/NIR‐treated group. Quantitative analysis revealed markedly reduced expression of these pro‐inflammatory cytokines compared to other groups (Figure S44, Supporting Information). These results demonstrate that the AIE NPs@BP NSs@FSs/NIR platform modulates key cytokines by upregulating IL‐10/TGF‐β and downregulating TNF‐α/IFN‐γ, thus shifting macrophage polarization from the M1 to the M2 phenotype. This immunomodulatory action helps alleviate local inflammation and fosters a regenerative microenvironment that supports bone defect repair.

To further investigate the biological mechanisms of the AIE NPs@BP NSs@FSs/NIR platform, we conducted RNA‐seq analysis. First, we compared gene expression levels under various experimental conditions (Figure S45, Supporting Information). Transcriptomic profiling identified 4954 upregulated and 3814 downregulated DEGs in the AIE NPs@BP NSs@FSs/NIR therapeutic group versus untreated controls. The upregulated DEGs were also demonstrated by GO enrichment analysis to be strongly linked to inflammatory regulatory pathways, antimicrobial defense, angiogenesis, cell adhesion, and osteogenesis, indicating that the implanted materials influence the immune microenvironment while promoting tissue regeneration (Figure 8E). KEGG pathway analysis supported these findings, revealing notable enrichment in signaling pathways such as Ras, PI3K‐Akt, Cell adhesion molecules, ECM‐receptor interaction, and Wnt (Figure 8F). Collectively, these results suggest that the AIE NPs@BP NSs@FSs/NIR platform exerts a complex biological effect, simultaneously reducing inflammation and infection while promoting vascularization, cell recruitment, and new bone formation through modulation of key signaling pathways.

## 3 Conclusion

In summary, we successfully prepared a new multifunctional platform loaded with both BP NSs and AIE NPs, which exhibit a photothermal effect to enhance the repair of infected bone defects. The AIE NPs@BP NSs@FSs showed excellent mechanical properties, good biosafety, and sustained phosphorus release. Thanks to the outstanding photothermal properties and high conversion efficiency of AIE NPs, the bacterial biofilm can be effectively destroyed under 1064 nm laser irradiation. Simultaneously, BP NSs degraded into PO_4_^3−^ in the body, promoting biomineralization. in vitro studies demonstrated that AIE NPs@BP NSs@FSs had angiogenic and osteogenic effects on MAECs and BMSCs, respectively. The anti‐inflammatory effect was further mediated by the AIE NPs@BP NSs@FSs platform, which promoted macrophage repolarization from M1 to M2 via ROS elimination and subsequent regulation of pro‐inflammatory gene expression. in vivo experiments on rat cranial infected bone defects confirmed that AIE NPs@BP NSs@FSs/NIR possessed effective photothermal antimicrobial properties, anti‐inflammatory effects, vasculogenic promotion, and osteogenic activity. These results demonstrate the superior efficacy of the multifunctional AIE NPs@BP NSs@FSs/NIR membrane in repairing infected bone defects, highlighting its promising potential for clinical translation.

## 4 Experimental Section

### Materials

The BP bulks, tetrahydrofuran (THF), and acetic acid (HAc) were purchased from Sinopharm Group Chemical Reagent Co., Ltd. The polycaprolactone (PCL), whose nominal molecular weight is 80 000 kDa, was procured from Sigma–Aldrich. The phospholipid polyethylene glycol amino (DSPE‐PEG_2000_) was purchased from Pengshuo Biology. Aladdin Reagent Company supplied both gelatin and 2,2,2‐trifluoroethanol (TFE, 99.5%). Dojindo Molecular Technologies and Sigma–Aldrich Trading Co., Ltd. (Shanghai, China) were the respective commercial sources for the Cell Counting Kit‐8 (CCK‐8) and the reagents alkaline phosphatase (ALP) and alizarin red S (ARS). Key reagents, such as the ROS analysis kit, PBS (pH 7.4), LB liquid medium, DMEM, and FBS, were procured from Jiangsu KeyGEN Bio‐Tech Corp., Ltd.

### Synthesis of BP NSs@AIE NPs@FSs

To obtain BP NSs, a liquid exfoliation procedure was employed. Initially, 100 mg of bulk BP was dispersed in 100 mL of deionized water and exfoliated via 12 h of ultrasonication on an ice bath. The crude suspension was first purified by centrifugation at 1000 rpm for 10 min to sediment any remaining large particles. The supernatant was then further centrifuged at 3800 rpm for 30 min to harvest the exfoliated nanosheets. The supernatants were collected after both centrifugations and stored at 4 °C in the dark. To prepare AIE NPs, 2 mg of DSPE‐PEG_2000_ and 1 mg of AIEgens were dissolved in 1 mL of THF. This solution was added dropwise into 9 mL of deionized water under sonication for 5 min.^[^ ^37^ ^]^ For fiber production, PCL and gelatin were dissolved in HFP; BP NSs and AIE NPs were then added and mixed for 24 h. Finally, the blend was supplemented with acetic acid and stirred for another hour. The final concentrations of BP NSs and AIE NPs were 200 and 100 µg mL^−1^, respectively. Using a voltage of 16 kV, a collector distance of 15 cm, and a flow rate of 1.5 mL h^−1^, fibers were electrospun onto aluminum foil and then dried. The specific formulations were provided in Table S1 (Supporting Information).

### Characterization

The morphology and size of AIE NPs and BP NSs were analyzed using TEM (FEI Tecnai G2) and DLS (Malvern Nano‐ZS). Fiber structure and diameter were examined through SEM and ImageJ. UV–vis and fluorescence spectra were recorded with a Shimadzu 3100 and FluoroMax‐4 (Hitachi), respectively. FTIR (Nicolet 200) and TGA (METTLER TOLEDO) assessed chemical structure and thermal stability. Zeta potential was measured using a SurPASS analyzer (Anton Paar). Surface composition was determined via XPS (Kratos AXIS Ultra DLD). Mechanical properties were tested with an Instron 3365 on dumbbell‐shaped samples (4 × 1 cm) at a rate of 10 mm min^−1^. Contact angles were measured with a goniometer. Degradation was evaluated through SEM and PBS‐based weight loss over 21 d.

### In Vitro Photothermal and Degradation Performance

To monitor temperature changes of AIE NPs and BP NSs aqueous dispersions (0–100 µg mL^−1^) under 1064 nm laser irradiation at varying power densities (0.25–1.0 W cm^2^, 10 min), an infrared thermal camera (FLIR ONE Pro) was used to collect temperature data and thermal images. AIE NPs@BP NSs@FSs were tested using the same method above. Thermal images and temperature curves were recorded every 30 s. Photothermal conversion efficiency (*η*) was calculated based on previously reported equations, incorporating parameters such as Tmax, absorbance at 1064 nm (A_1064_), laser power (I), and system time constant (τ):

(1) $$\eta =\frac{\mathrm{hs}\left(T_{\max}-T_{\mathrm{sur}}\right)-Q_{\mathrm{dis}}}{I(1-10^{-A_{1064}})}$$

(2) $$\mathrm{hs}=\frac{C_{s}m_{s}}{\tau }$$

(3) $$\tau =-\tau_{s}\ln\theta$$

(4) $$\theta =\frac{\Delta T}{\Delta T_{\max}}$$

Photothermal stability was assessed by five cycles of laser irradiation (1.0 W cm^2^, 10 min) and natural cooling for both AIE NPs and AIE NPs@BP NSs@FSs.

To evaluate degradation, AIE NPs@BP NSs@FSs were incubated at 37 °C and exposed to 1064 nm laser irradiation (1.0 W cm^2^, 10 min) three times daily. At days 0, 3, 7, 14, and 21, the release of phosphate ions in the supernatant was quantified using a phosphate assay kit (BioAssay Systems).

### Total Antioxidant Capacity Assay

The antioxidant capability of BP NSs and AIE NPs@BP NSs@FSs was assessed using DPPH and ABTS radical scavenging assays. A standard DPPH assay was performed. Samples containing BP NSs (0–100 µg mL^−1^) were mixed with 0.1 mmol L^−1^ DPPH in ethanol and incubated in the dark at 37 °C for 40 min. The absorbance at 517 nm was monitored at 10 min intervals. The scavenging rate was then computed using the formula:

(5) $$\mathrm{Scavenging}\;\mathrm{Rate}=\left(1-\frac{A_{t}}{A_{i}}\right)\;\times 100\%$$

where A_i_ is the initial absorbance and A_t_ is the absorbance after 20 min.

For the ABTS assay, oxidized ABTS·⁺ solution was reacted with the same sample concentrations. After incubation for 20 min, the absorbance at 734 nm was measured. Scavenging efficiency was calculated by:

(6) $$\mathrm{ABTS}\;\mathrm{scavenging}\;\mathrm{ratio}\;\left(\%\right)=\frac{A_{\mathrm{control}}-A_{\mathrm{sample}}}{A_{\mathrm{control}}}\times 100$$

where A_control_ is the absorbance of the blank and A_sample_ is the absorbance after reaction.

### In Vitro Photothermal Antibacterial Performance

The antibacterial effectiveness of the fibrous membranes against *S. aureus* (ATCC 25 923) and *E. coli* (ATCC 25 922) was evaluated using several in vitro assays. Membranes were incubated with bacterial suspensions (1 × 10⁷ CFU mL^−1^) at 37 °C for 8 h, then subjected to NIR‐II irradiation (1064 nm, 1.0 W cm^2^, 10 min). The treated suspensions were serially diluted and plated for CFU counting. Bacterial viability was assessed with SYTO9/PI dual staining. Imaging of the stained samples was performed with an inverted fluorescence microscope. Morphological changes were observed using SEM. The main experimental steps involved 2.5% glutaraldehyde fixation, gradient ethanol dehydration, and slide sealing with silicon. Following staining with SYTO9/PI, the biofilms were examined using a confocal laser scanning microscope (CLSM; ZEISS LSM 880). Biomass was quantified with crystal violet staining, and absorbance at 590 nm was measured after solubilization in 33% acetic acid. Total RNA was isolated using RNAiso Plus. Reverse transcription and qPCR were performed with Takara kits, using 16S rRNA as the internal control. Primer sequences were listed in Table S2 (Supporting Information).

### Cell Culture

L929 (RRID: CVCL_0462) and RAW 264.7 (RRID: CVCL_0493) cells were purchased from the Type Culture Collection of the Chinese Academy of Sciences (Shanghai, China). Mouse aortic endothelial cells (MAECs; Cat# CL0200, Fenghui Biotech) and human oral keratinocytes (HOKs; Cat# PRI‐H‐00140, Zhongqiao Xinzhou Biotech) were used as primary cells. All cells were confirmed mycoplasma‐free and cultured in DMEM supplemented with 10% FBS and 1% penicillin/streptomycin. Rat bone marrow‐derived mesenchymal stem cells (BMSCs) were isolated from 3–5‐day‐old SD rats. After euthanasia and disinfection, the bone marrow was harvested from the femurs and tibias by flushing it into a complete α‐MEM medium (Gibco, USA) containing 10% FBS and 1% penicillin/streptomycin. For osteogenic induction, BMSCs were cultured in α‐MEM supplemented with 50 µg mL^−1^ ascorbic acid, 10 mM β‐glycerophosphate, and 10 nM dexamethasone (Sigma–Aldrich), with medium refreshed every 2 d. All cultures were kept at 37 °C in a 5% CO_2_ atmosphere.

### Cytocompatibility, Proliferation, and In Vitro Anti‐Inflammatory/Angiogenic Evaluations

To assess cytocompatibility, L929 and HOK cells (2 × 10⁵ cells/20 µL) were seeded on fibrous membranes and incubated for 24 h. Live/Dead staining was used to visualize cell viability through fluorescence microscopy. A density of 8000 cells per well was seeded into 96‐well plates and co‐cultured with the test membranes. Following incubation for 24, 48, and 72 h, cell viability and proliferation were analyzed via the CCK‐8 assay. Absorbance readings at 450 nm were used to determine relative viability and to plot the proliferation curves. For the anti‐inflammatory studies, RAW 264.7 macrophages were first activated with LPS (10 µg mL^−1^) in a hypoxic environment (1% O_2_) before a 24 h incubation with the different membrane samples. Cytokine expression was analyzed by immunofluorescence, flow cytometry, and qPCR. Primer sequences were provided in Table S3 (Supporting Information). Cell migration was evaluated using a scratch assay in MAECs and L929 cells, with wound closure monitored over 8 h. In angiogenesis assays, pretreated MAECs were seeded on Matrigel‐coated plates (1 × 10⁵ cells well^−1^), incubated for 8 h, and tube formation was imaged and quantified with ImageJ based on total length and branch points.

### Osteogenic and Osteoclastogenic Evaluation

To assess osteogenesis in an inflammatory microenvironment, BMSCs were cultured under different conditions. ALP activity and staining were evaluated on day 7, and mineralization was assessed by ARS staining on day 21. Stain quantification was performed by measuring absorbance at 562 nm. Cytokine expression was analyzed by qPCR. Mature osteoclasts were identified as multinucleated (≥3 nuclei), TRAP‐positive cells. To generate them, RAW264.7 cells were cultured for 3 days under LPS‐induced hypoxia in the presence of M‐CSF and RANKL before being stained with a TRAP kit. The primer sequences used can be found in Table S4 (Supporting Information).

### Western Blot Analysis

Western blotting was performed to assess the expression and phosphorylation of key NF‐κB pathway proteins (IκBα and NF‐κB p65) in RAW264.7 cells following LPS stimulation and subsequent treatments. SDS‐PAGE separated proteins, transferred to PVDF membranes, and probed with rabbit anti‐NF‐κB p65 (Zenbio, 380 172), anti‐phospho‐NF‐κB p65 (CST, 3033), anti‐IκBα (Zenbio, 380 682), anti‐phospho‐IκBα (Zenbio, 340 776), and anti‐GAPDH (CST, 2118), all diluted 1:1000. Chemiluminescence detection was used to visualize the immunoreactive bands, using an HRP‐linked goat anti‐rabbit IgG (1:2000, CST, 7074) as the secondary antibody.

### In Vivo Infective Bone Defect and Histological Assessment

All animal experiments were performed in accordance with the “Principles of Laboratory Animal Care” (NIH Publication No. 86‐23, revised 1985) and approved by the Institutional Animal Care and Use Committee (IACUC) of Nanjing Medical University (Approval No. IACUC‐2412001). A 5 mm full‐thickness calvarial defect was created in 4‐week‐old male SD rats (100–150 g), followed by inoculation with 10 µL of *S. aureus* (1 × 10⁷ CFU mL^−1^) to induce infection. Rats were randomly assigned to five treatment groups. On day 3, wound exudates were collected for CFU counting. At weeks 4 and 8, calvaria were collected, fixed, and examined via micro‐CT. Bone regeneration was quantified through 3D reconstruction, including BV/TV, bone area, and Tb.N. Prior to H&E and Masson's trichrome staining, the samples were decalcified, embedded in paraffin, and sectioned. In separate immunofluorescence experiments, sectioned tissues were exposed to anti‐CD80 and anti‐CD206 primary antibodies (1:100, Santa Cruz) in a 4 °C overnight incubation. Subsequently, they were treated with the appropriate secondary antibodies (1:100, Santa Cruz) for one hour at room temperature. Additional sections underwent Gram staining and immunohistochemistry (IHC) targeting OCN (TD12303, Abmart, China), CD31 (ab281583, Abcam, USA), IFN‐γ (PY6252S, Abmart, China), IL‐10 (60269‐1‐lg, Proteintech, China), TGF‐β (21898‐1‐AP, Proteintech, China), and TNF‐α (ab307164, Abcam, USA). Slides were examined under an optical microscope.

### Statistical Analysis

All statistical analyses were performed using GraphPad Prism 7.0 (GraphPad Software, USA). Normality and outliers were assessed using the Shapiro–Wilk test. Data were presented as mean ± standard deviation (s.d.), with *n* = 3 unless otherwise specified. For comparisons among multiple groups, one‐way or two‐way ANOVA was applied as appropriate, followed by Tukey's post hoc test. Unpaired two‐tailed Student's t‐tests were used for pairwise comparisons. Survival data were analyzed using the log‐rank (Mantel–Cox) test. Statistical significance was defined as *p < 0.05, **p < 0.01, and ***p < 0.001.

## Conflict of Interest

The authors declare no conflict of interest.

## Supporting information



Supporting Information

## Data Availability

The data that support the findings of this study are available from the corresponding author upon reasonable request.
