# Supplementary material for: NIR‐II Responsive Multifunctional Scaffold Enabling “Kill‐Modulation‐Build” Synergistic Therapy for Infectious Bone Defects
Source: Adv Sci (Weinh). 2025 Oct 7;12(47):e08948. doi: 10.1002/advs.202508948 (PMC12713057; doi:10.1002/advs.202508948)
Supplement: Supplementary file 1 — Supporting Information [file ADVS-12-e08948-s001.docx]

Supporting Information

**NIR-II Responsive Multifunctional Scaffold Enabling “Kill-Modulation-Build” Synergistic Therapy for Infectious Bone Defects**

**Supplementary Figures**


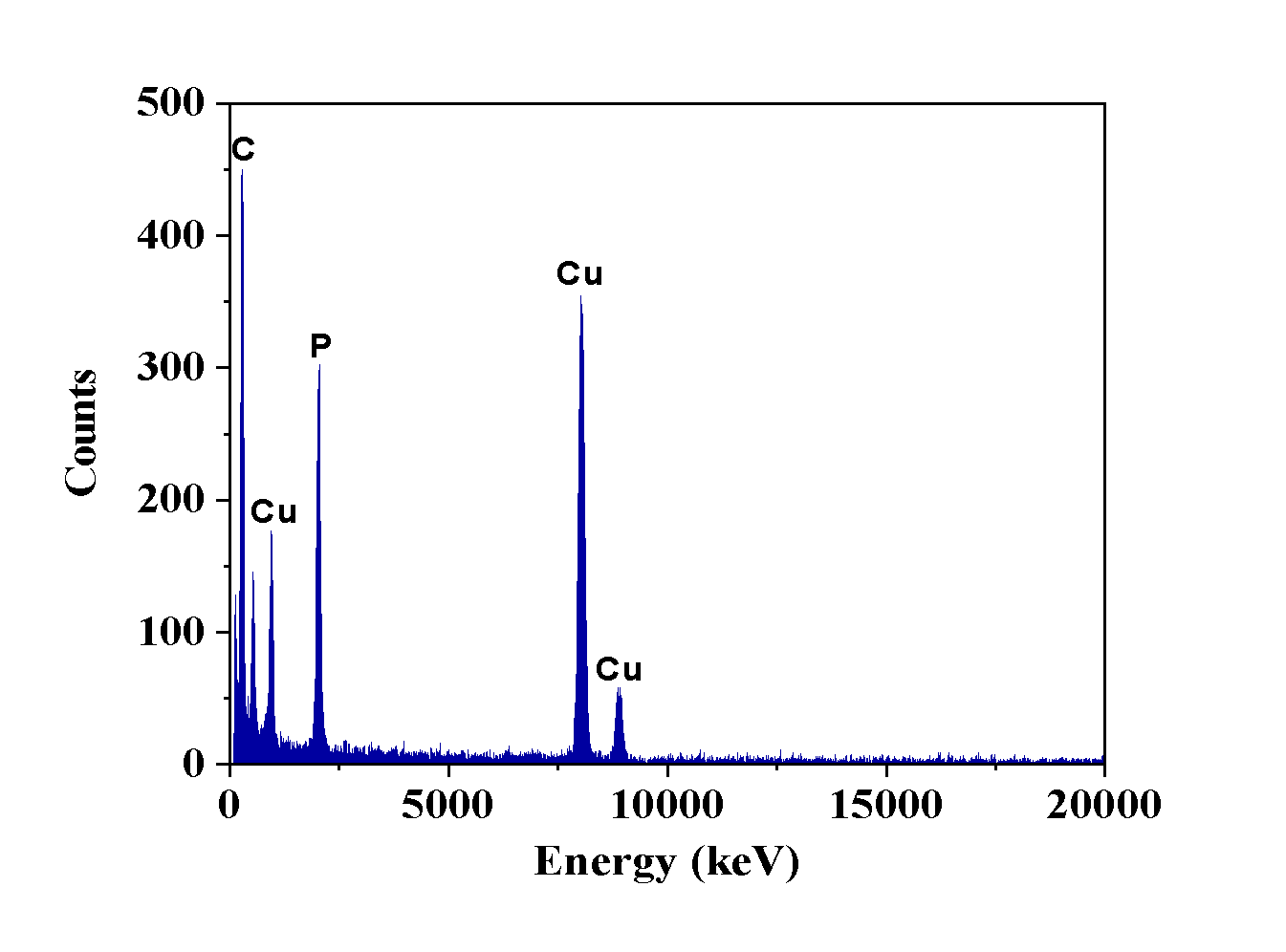


**Figure S1.** EDX spectrum of BP NSs.


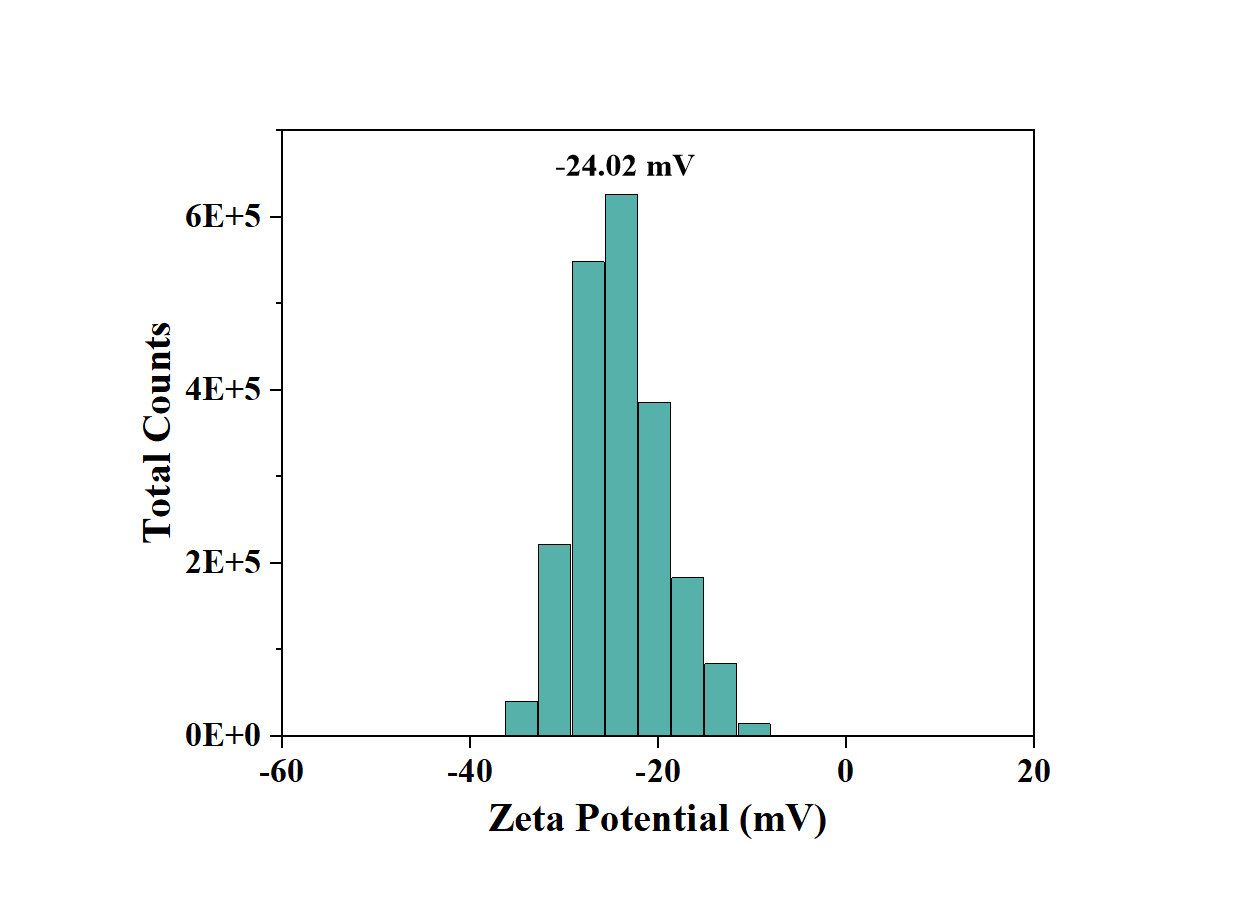


**Figure S2.** Zeta potential of AIE NPs.


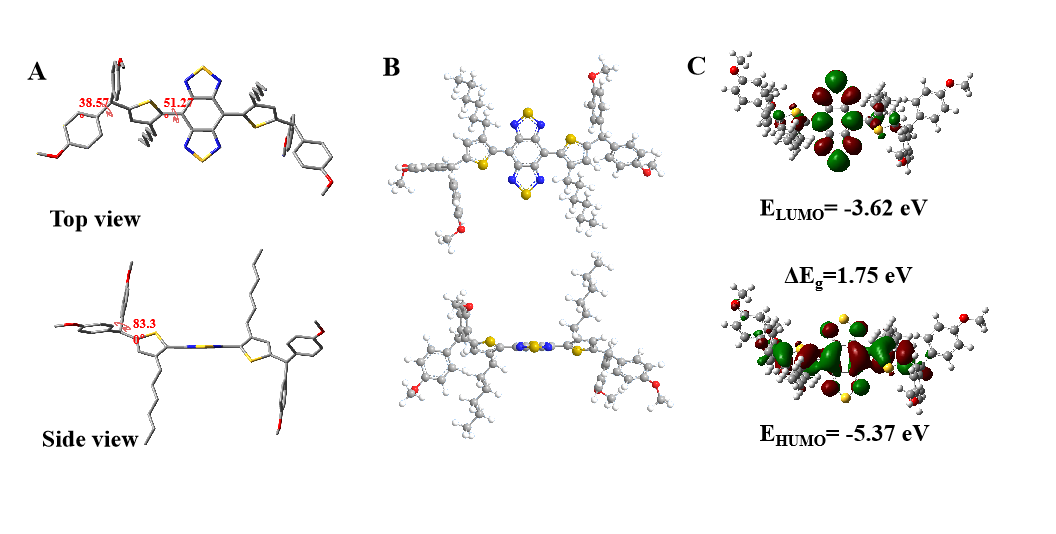


**Figure S3.** (A) Optimized molecular geometries (B), single crystal structure, and (C) HOMO-LUMO orbitals of AIEgens.


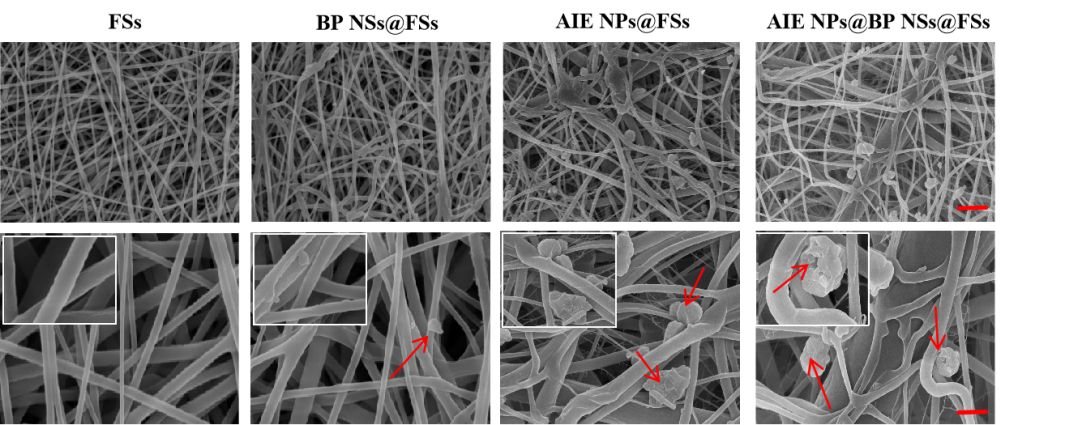


**Figure S4.** SEM images of four types of fibrous membranes at different magnifications. Scale bars, 1 μm (up) and 500 nm (down).


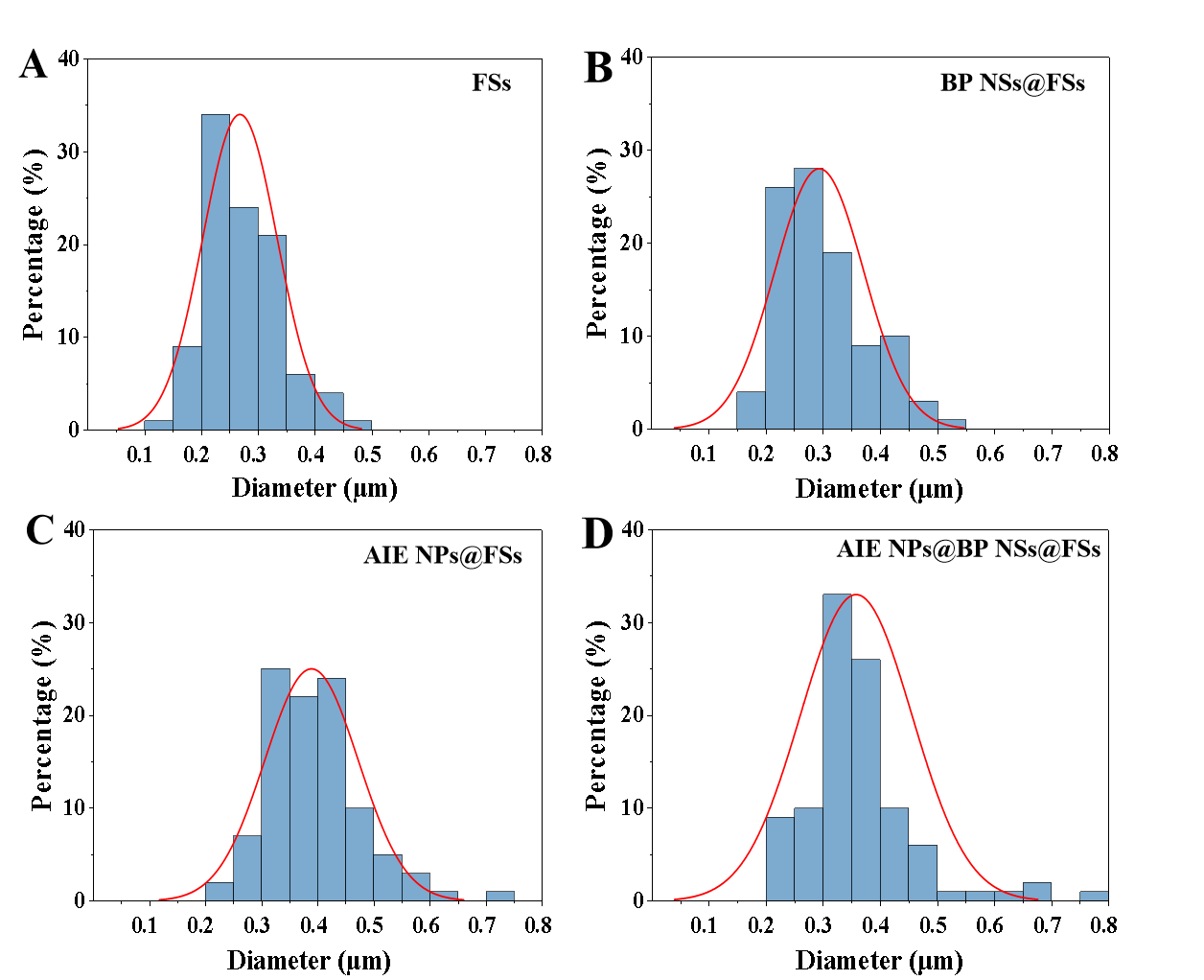


**Figure S5.** Histograms of (A) FSs, (B) BP NSs@FSs, (C) AIE NPs@FSs, and (D) AIE NPs@BP NSs@FSs fibrous diameters.


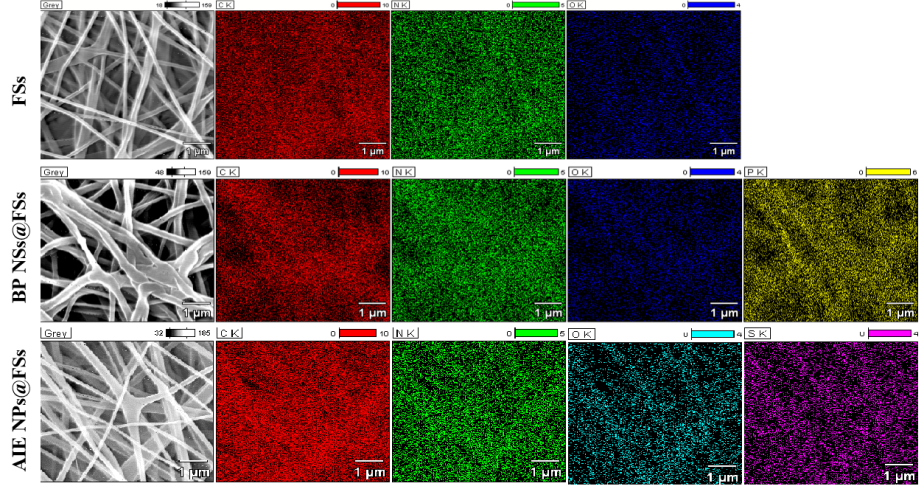


**Figure S6.** SEM images and elemental mapping of fibrous membranes. Scale bars, 1 μm.


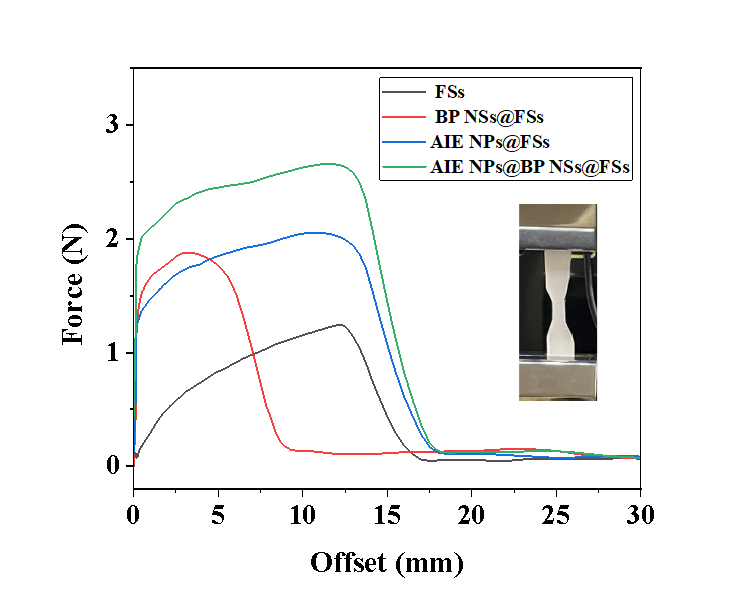


**Figure S7.** Tensile stress-strain curves of four types of fibrous membranes (samples were cut into 4×1 cm dumbbell shapes).


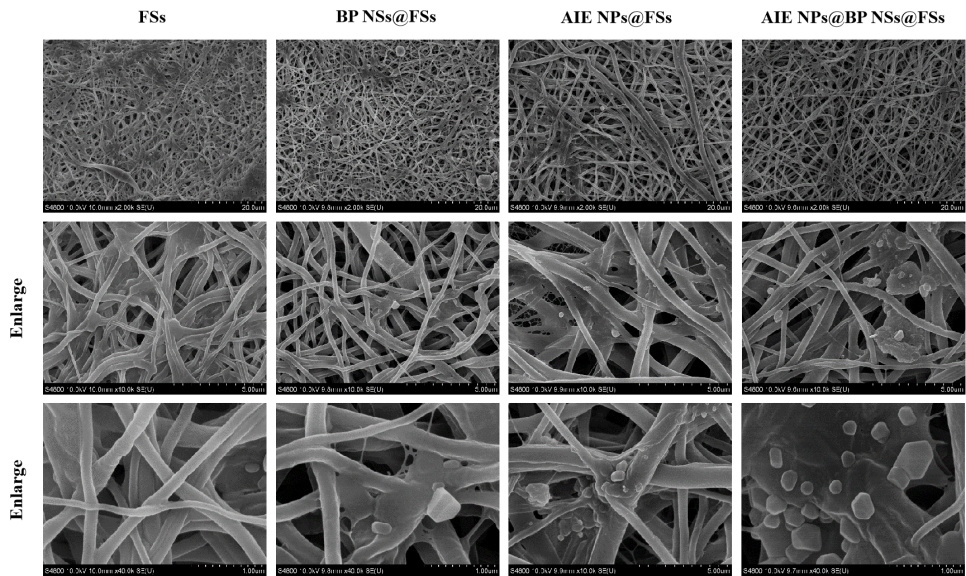


**Figure S8.** Degradation profiles and SEM images of four fibrous membranes on day 21. Scale bars, 20 μm (up), 5 μm (middle), or 1 μm (down).


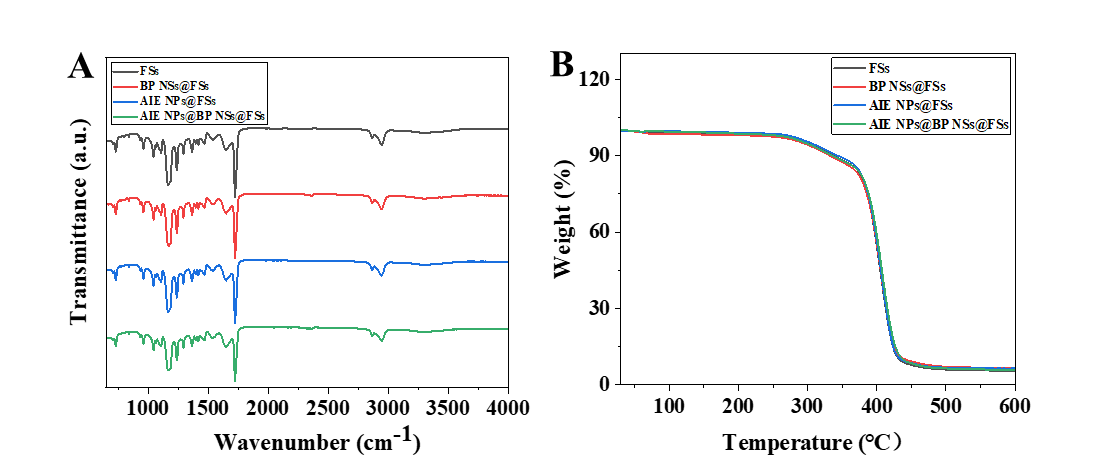


**Figure S9.** (A) FTIR spectra of fibrous membranes. (B) TGA of fibrous membranes.


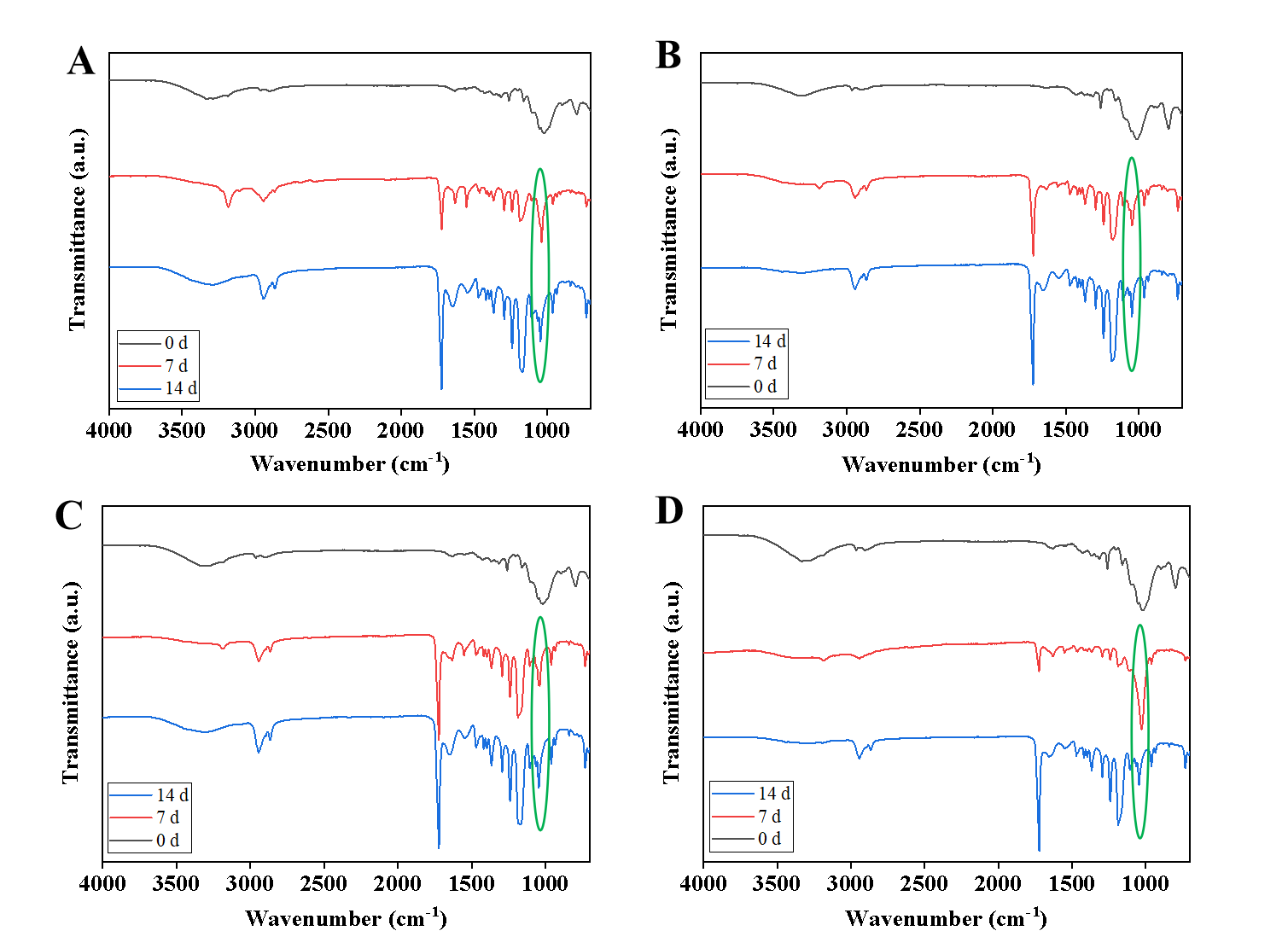


**Figure S10.** FTIR spectra of degradation fibrous membranes of (A) FSs, (B) BP NSs@FSs, (C) AIE NPs@FSs, and (D) AIE NPs@BP NSs@FSs on days 0, 7, and 14.


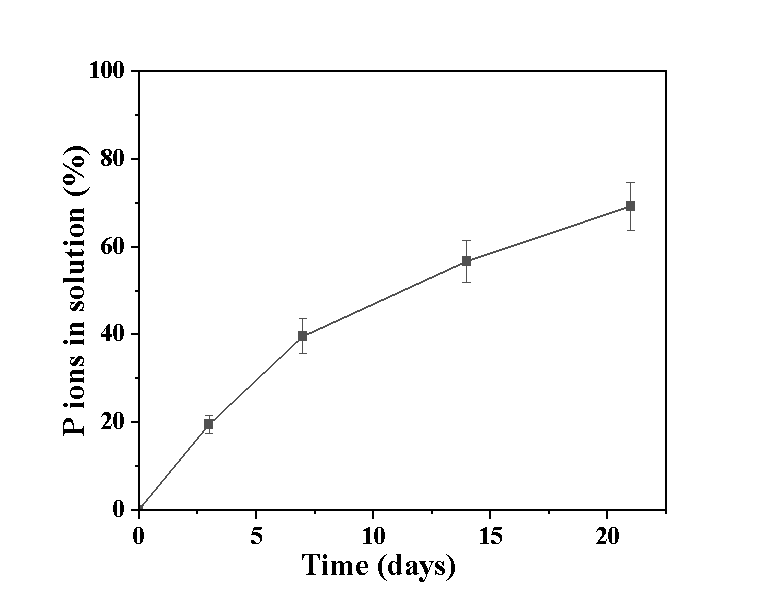


**Figure S11.** Cumulative phosphorus release concentration of AIE NPs@BP NSs@FSs in SBF solution on days 0, 3, 7, 14, and 21 (data presented as mean ± s.d., N = 3).


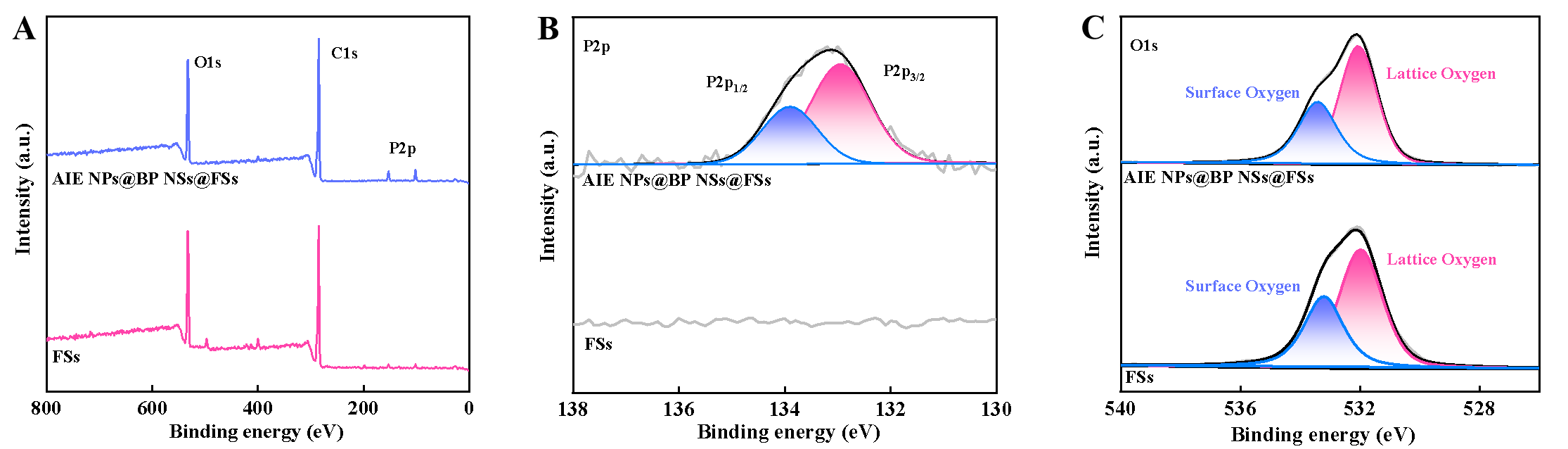


**Figure S12.** (A) X-ray photoelectron spectroscopy (XPS) spectra of AIE NPs@BP NSs@FSs and FSs. (B) XPS spectra of P 2p. (C) XPS spectra of O 1s.


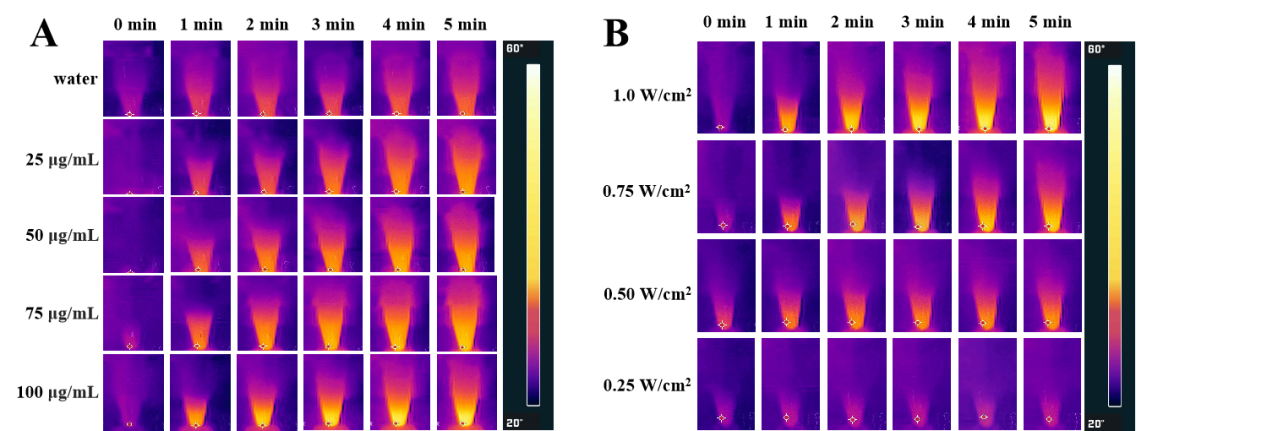


**Figure S13.** (A) Thermal images of AIE NPs at varying concentrations under a 1064 nm laser exposure. **(**B) Thermal images of 100 μg/mL AIE NPs at different power densities under a 1064 nm laser irradiation.


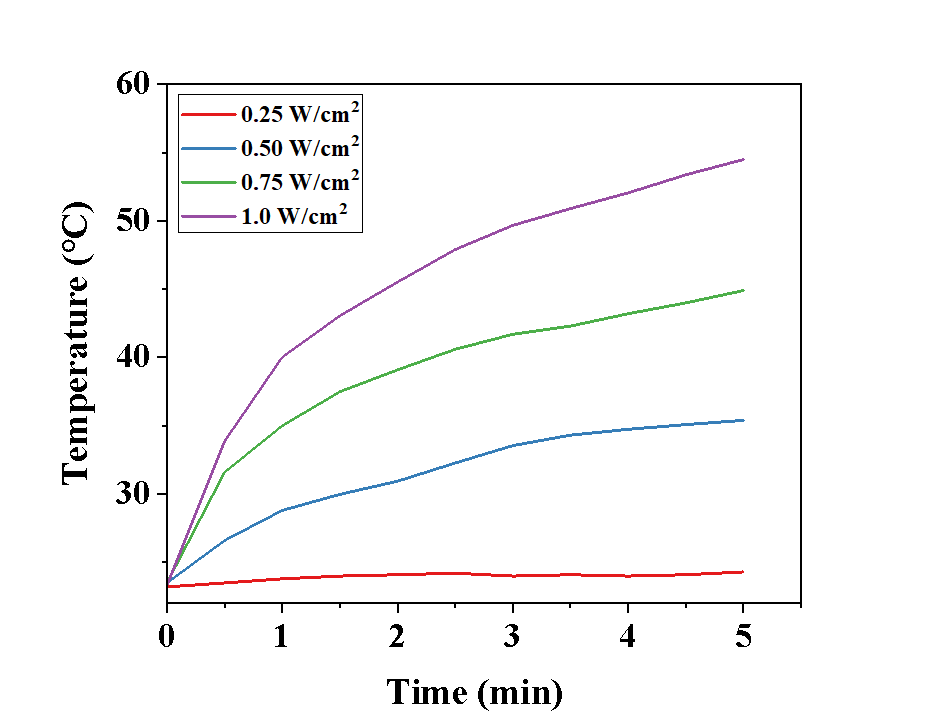


**Figure S14.** The temperature variation curve of AIE NPs at different power densities under a 1064 nm laser exposure.


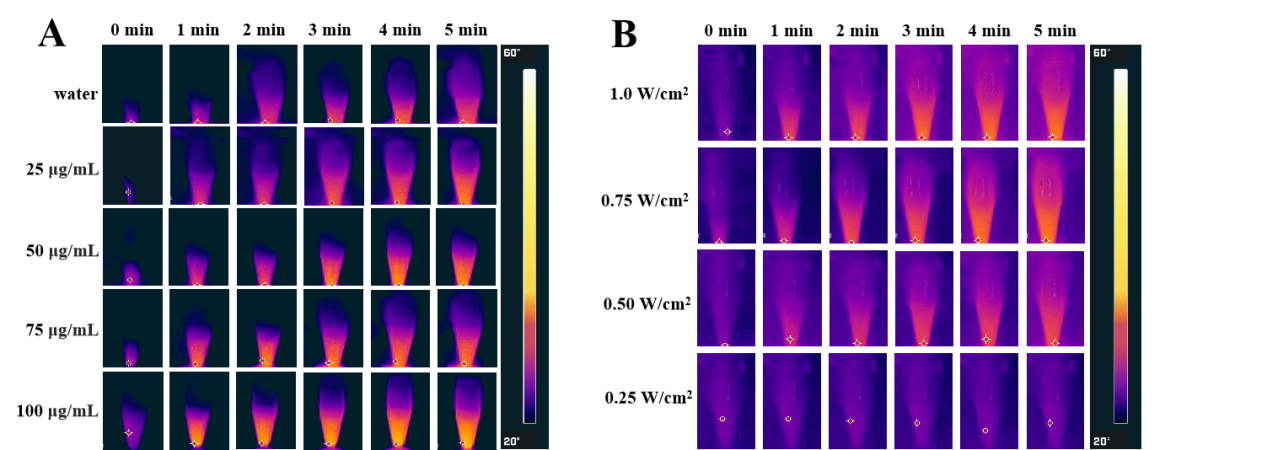


**Figure S15.** (A) Thermal images of BP NSs at varying concentrations under a 1064 nm laser exposure. **(**B) Thermal images of 100 μg/mL BP NSs at different power densities under a 1064 nm laser irradiation.


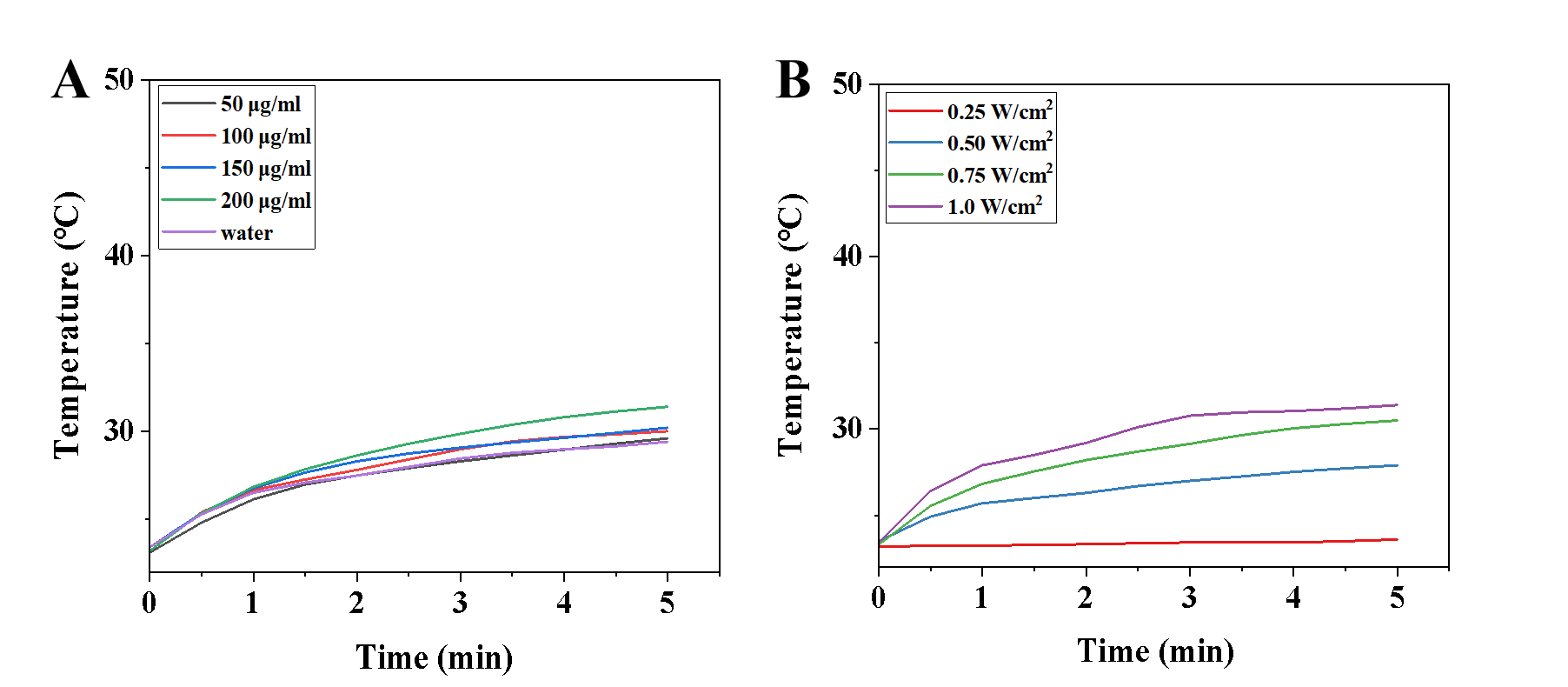


**Figure S16.** (A) The temperature variation curve of BP NSs at varying concentrations under 1064 nm laser exposure. (B) Temperature variation curves of BP NSs at different power densities under a 1064 nm laser exposure.


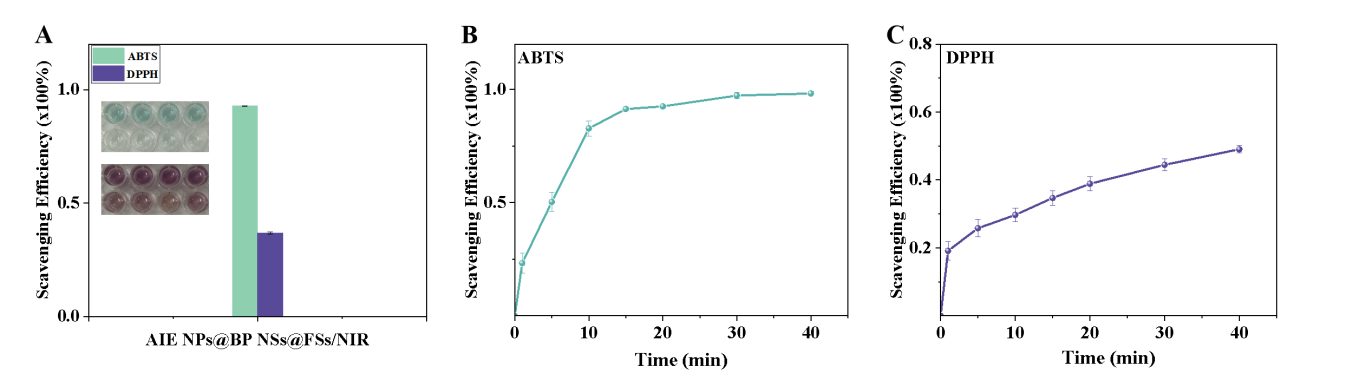


**Figure S17. (**A) Free radical scavenging rates of ABTS·^+^ and DPPH· treated with AIE NPs@BP NSs@FSs/NIR at 20 min. (B) Free radical scavenging kinetics of ABTS·^+^ treated with AIE NPs@BP NSs@FSs/NIR. (C) Free radical scavenging kinetics of DPPH· treated with AIE NPs@BP NSs@FSs/NIR (data presented as mean ± s.d., N = 3).


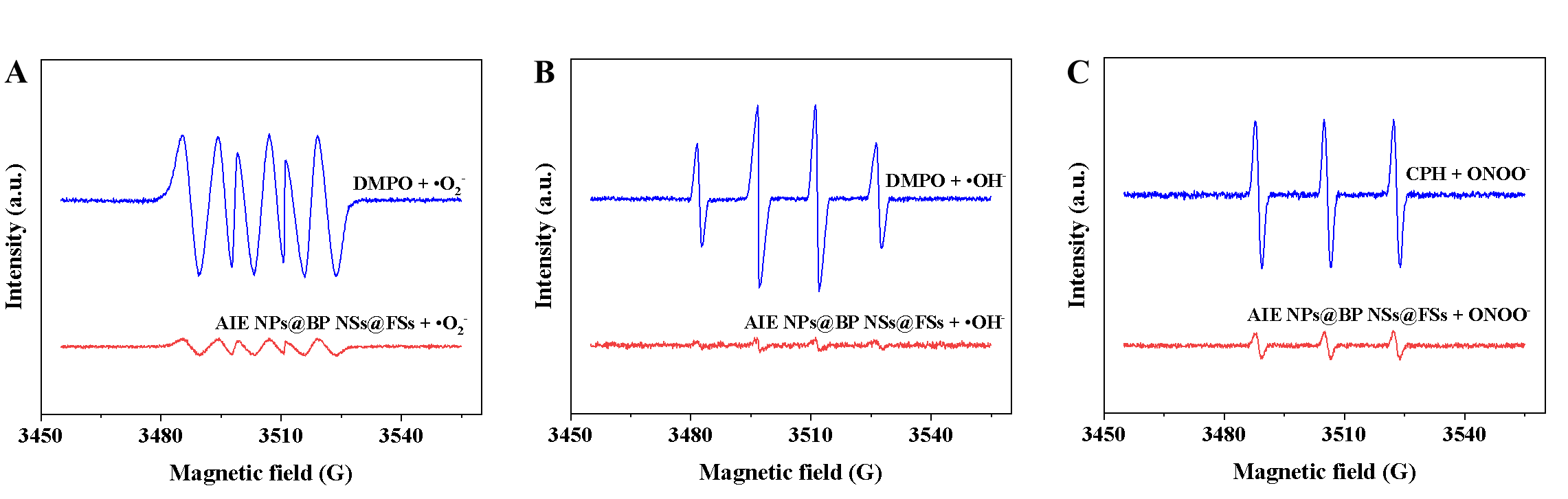


**Figure S18.** ESR measurement of (A) •O_2_⁻, (B) •OH⁻, and (C) ONOO⁻ scavenging capacity of AIE NPs@BP NSs@FSs.


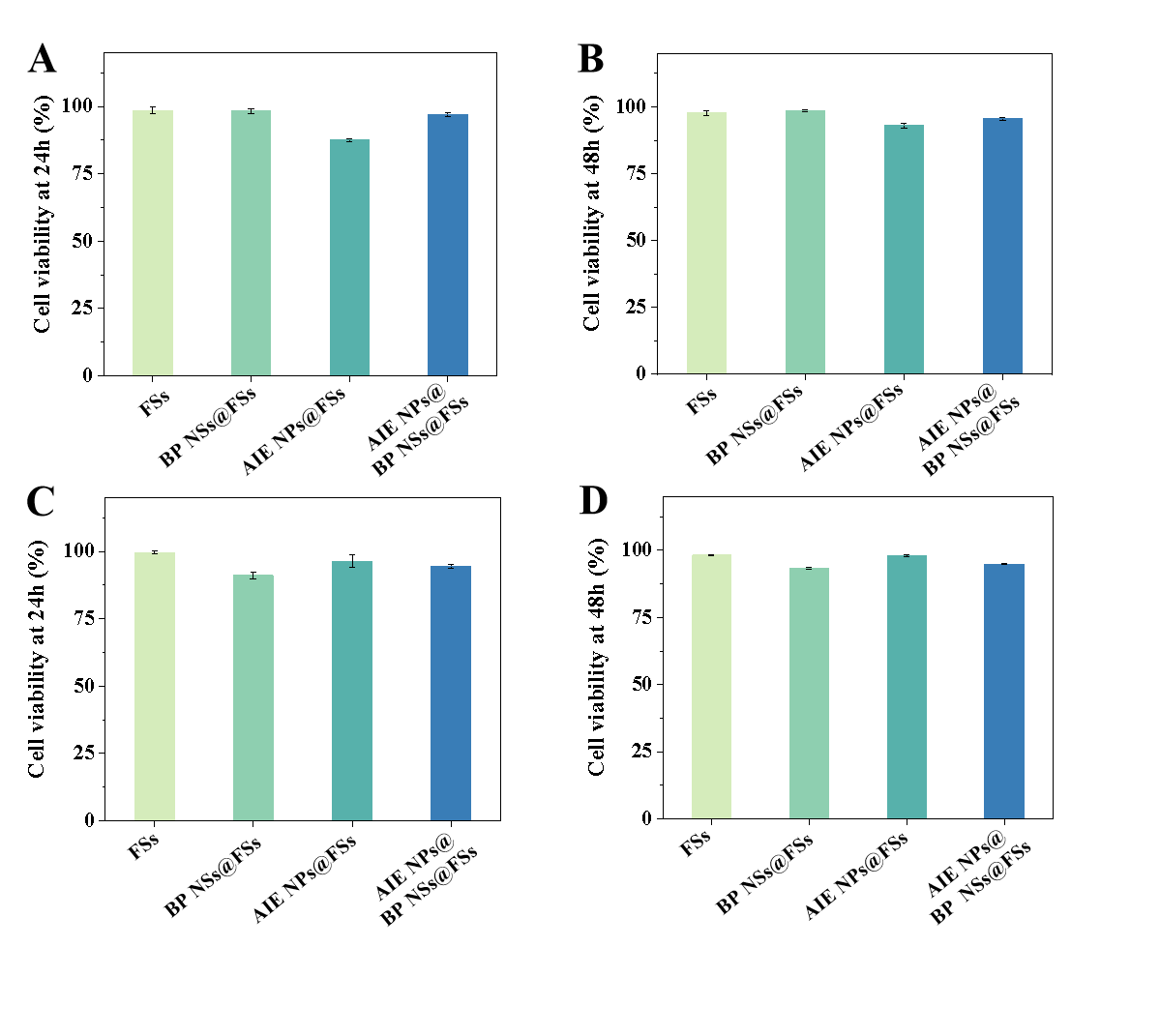


**Figure S19.** Cell viability of L929 cells at (A) 24h and **(**B) 48h with different fibrous membranes treatments. Cell viability of HOK cells at (C) 24h and **(**D) 48h with different fibrous membranes treatments (data presented as mean ± s.d., N = 3).


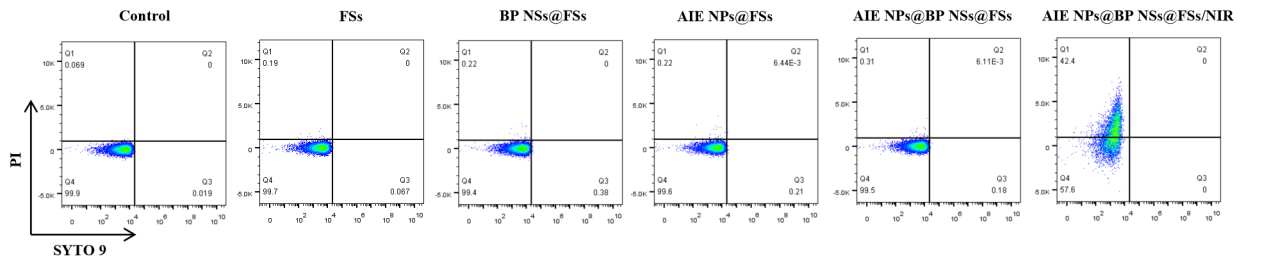


**Figure S20.** The flow cytometry analysis demonstrated variations in fluorescence intensity associated with *S. aureus* live/dead staining following various treatments.


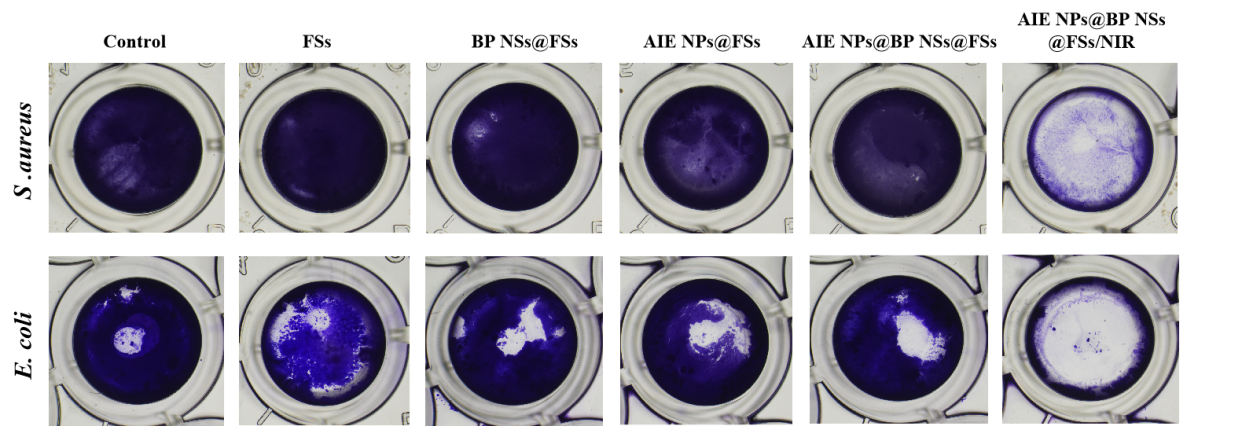


**Figure S21.** Crystal violet staining of biofilms formed by *S. aureus* and *E. coli* after different treatments.


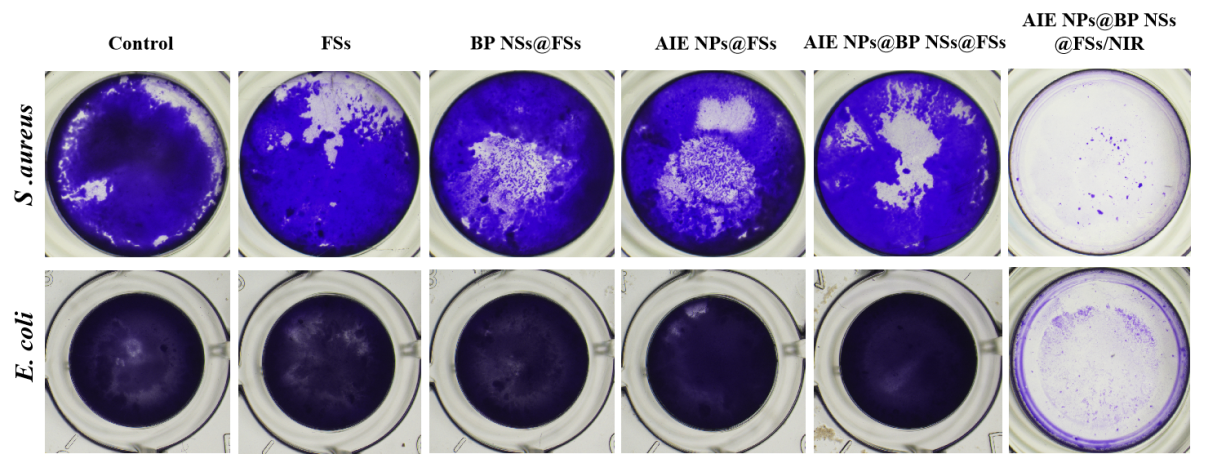


**Figure S22.** Crystal violet staining of biofilms formed by *E. coli* and *S. aureus* after different treatments.


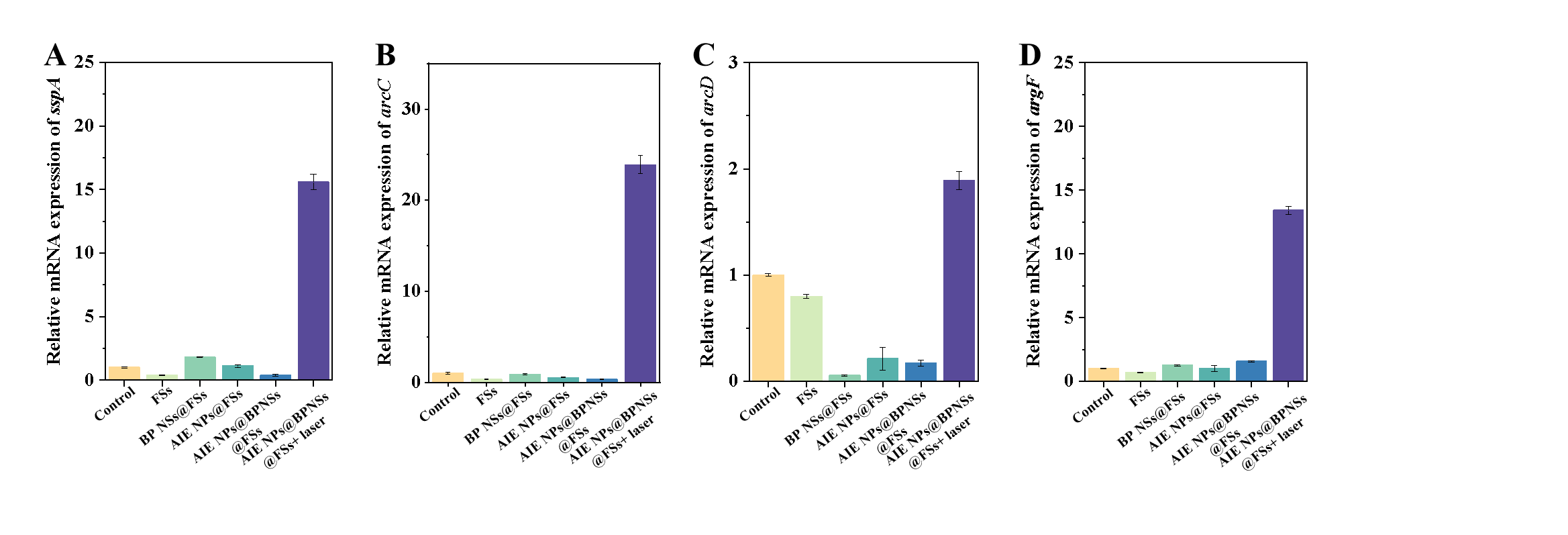


**Figure S23.** qPCR analysis of the expression levels of genes (A) *sspA,* (B) *arcC,* (C) *arcD,* and (D) *argF* related to inhibition of bacterial biofilm formation after different treatments. (data presented as mean ± s.d., N = 3).


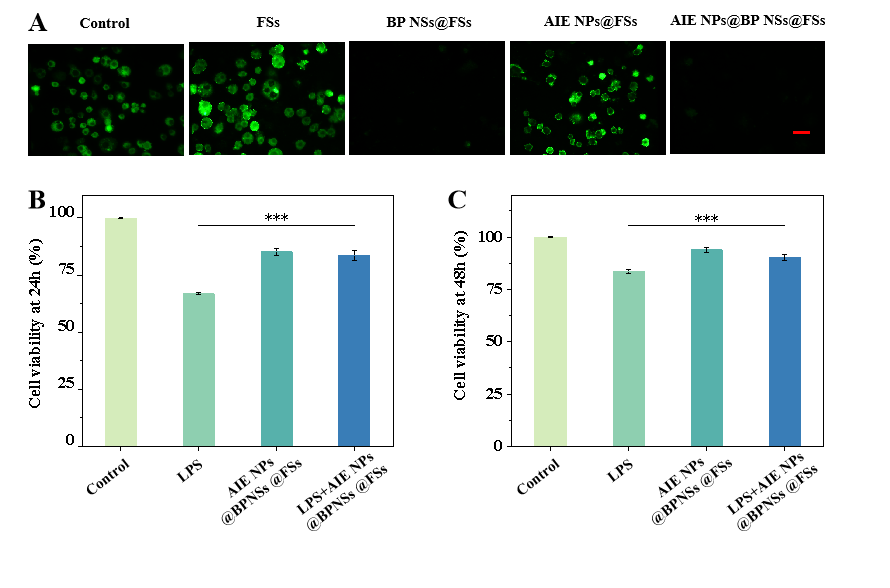


**Figure S24. (**A) ROS staining of RAW264.7 cells under different treatments. Scale bar, 20 μm. Cell viability of MAECs cell lines under different treatments for (B) 24h and (C) 48h. Data are shown as mean ± s.d. (statistical significances were calculated by *t*-test: ****p < 0.001*, N=3).


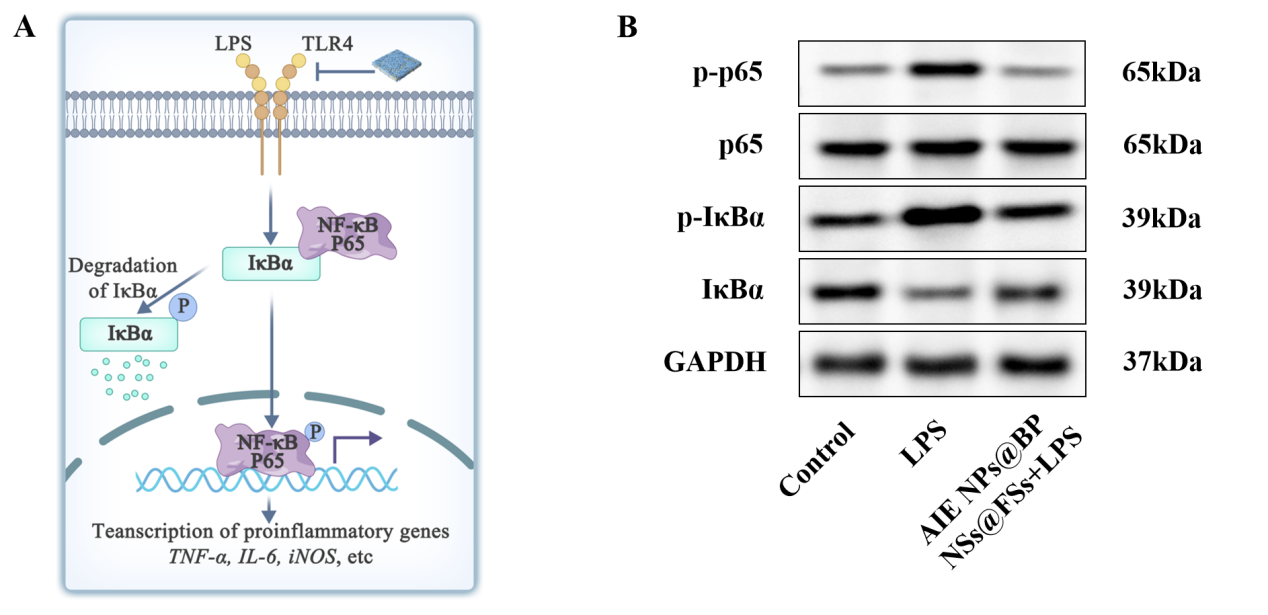


**Figure S25.** (A) The mechanism of AIE NPs@BP NSs@FSs inhibiting the activation of the NF-κB signaling pathway to restrain the proinflammatory state. (B) Western Blot of key protein expression levels (p-p65, p65, p-IκBα, and IκBα) in the NF-κB signaling pathway after different treatments in RAW264.7 cells for 24 h.


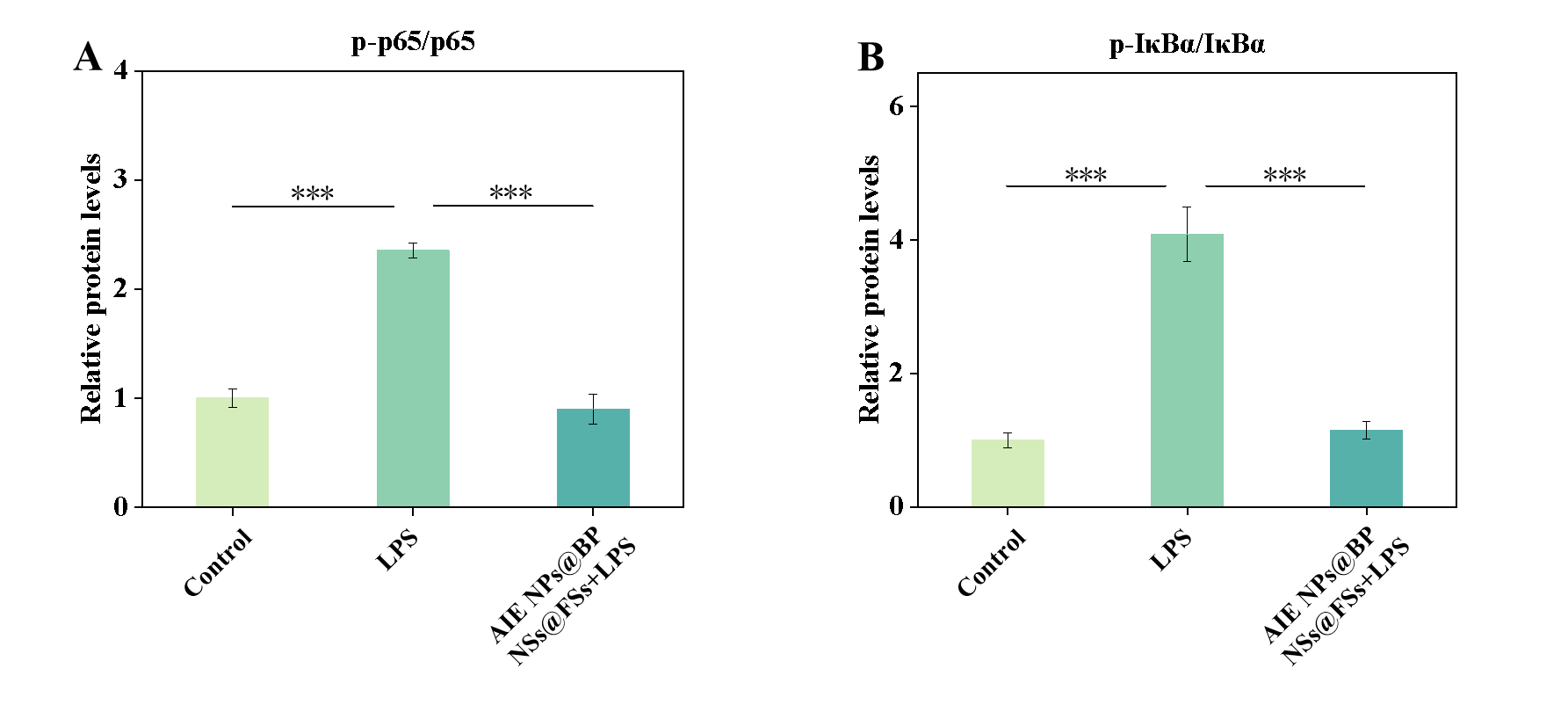


**Figure S26.** (A) Quantitative analysis of p-p65/p65 and (B) p-IκBα/IκBα expression in the NF-κB signaling pathway of RAW264.7 cells after 24 h of different treatments.


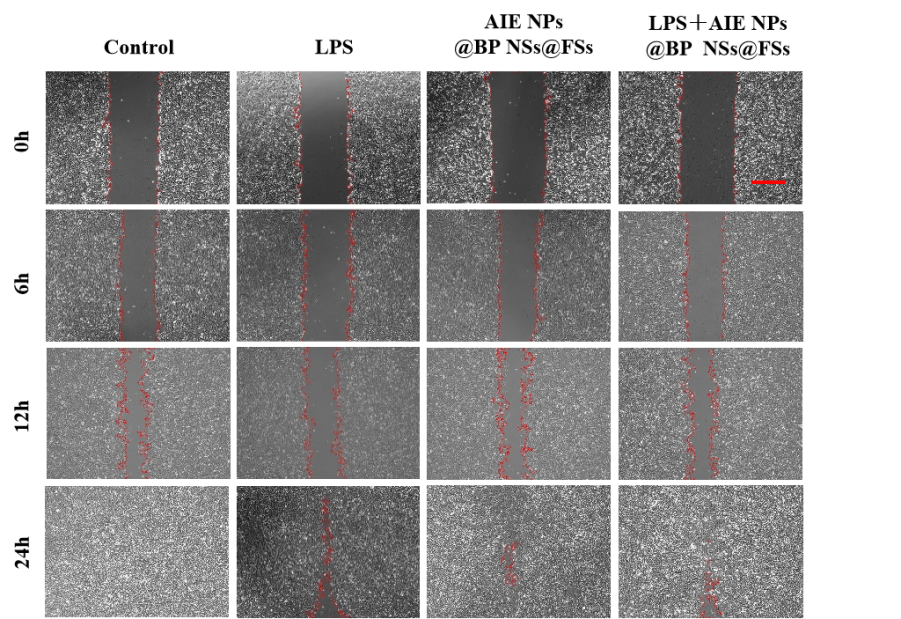


**Figure S27.** Representative images of the scratch migration assay of MAECs under different treatments. Scale bar, 100 μm.


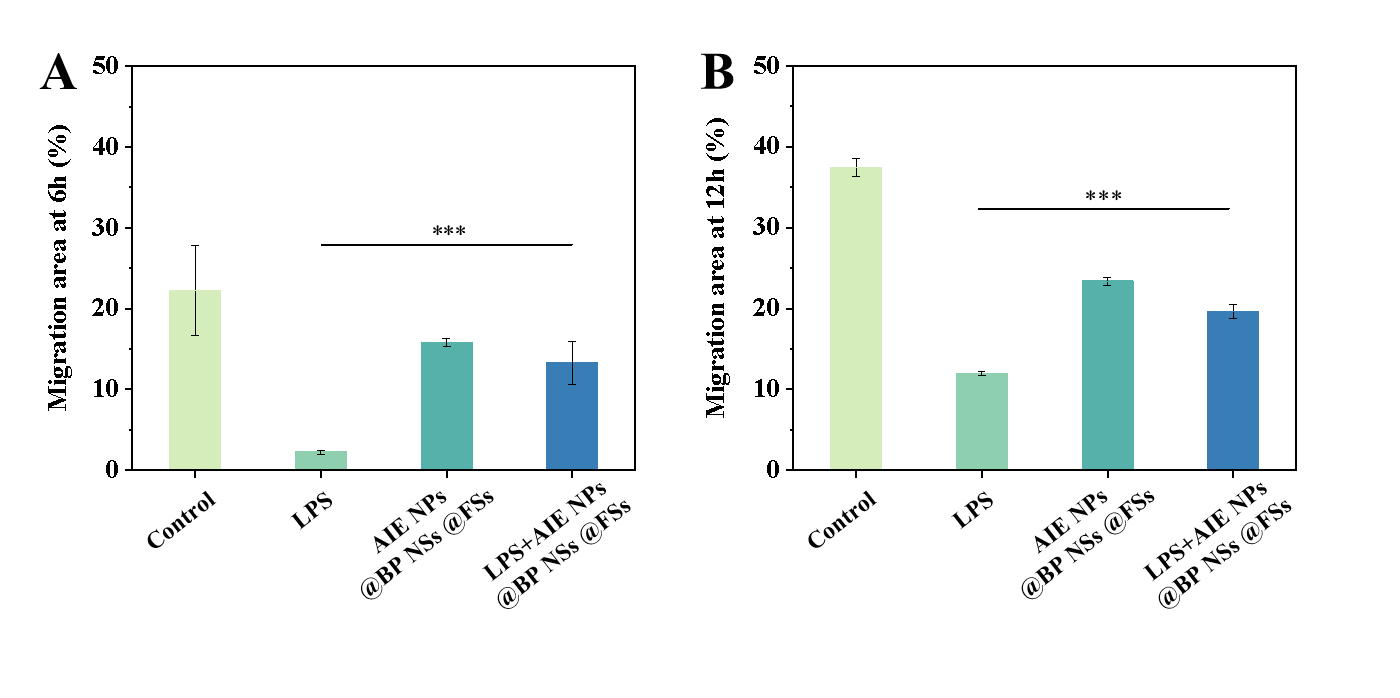


**Figure S28.** Quantitative analysis of scratch migration of the MAECs cell line under different treatments at (A) 6h, and (B) 12h. Data are shown as mean ± s.d. (statistical significances were calculated by *t*-test: ****p < 0.001*, N=3).


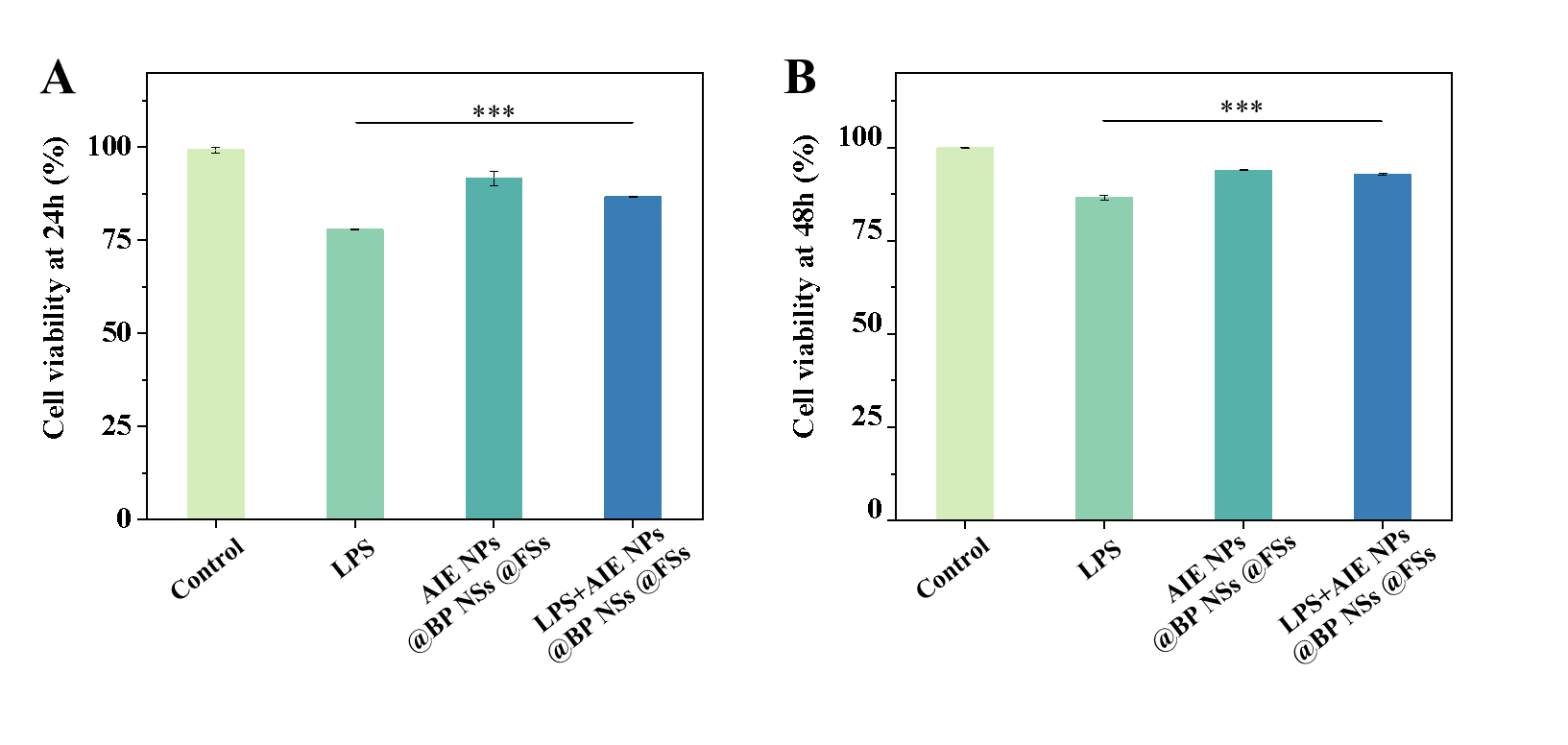


**Figure S29.** Cell viability of L929 cells under different membrane fiber treatments for (A) 24h and (B) 48h. Data are shown as mean ± s.d. (statistical significances were calculated by *t*-test: ****p < 0.001*, N=3).


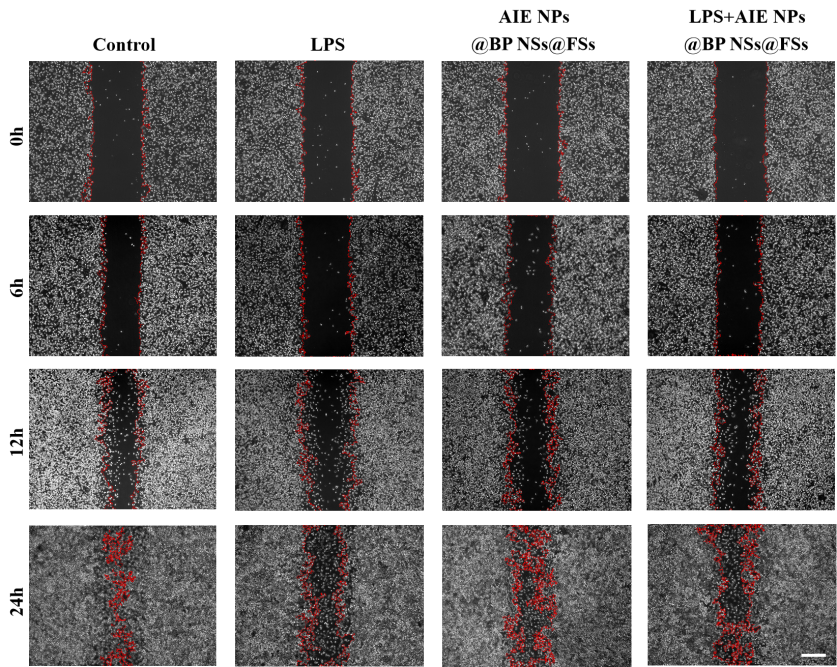


**Figure S30.** Representative images of the scratch migration assay of L929 cells under different treatments. Scale bar, 20 μm. Data are shown as mean ± s.d. (statistical significances were calculated by *t*-test: **p < 0.05, **p < 0.01, ***p < 0.001,* N=3).


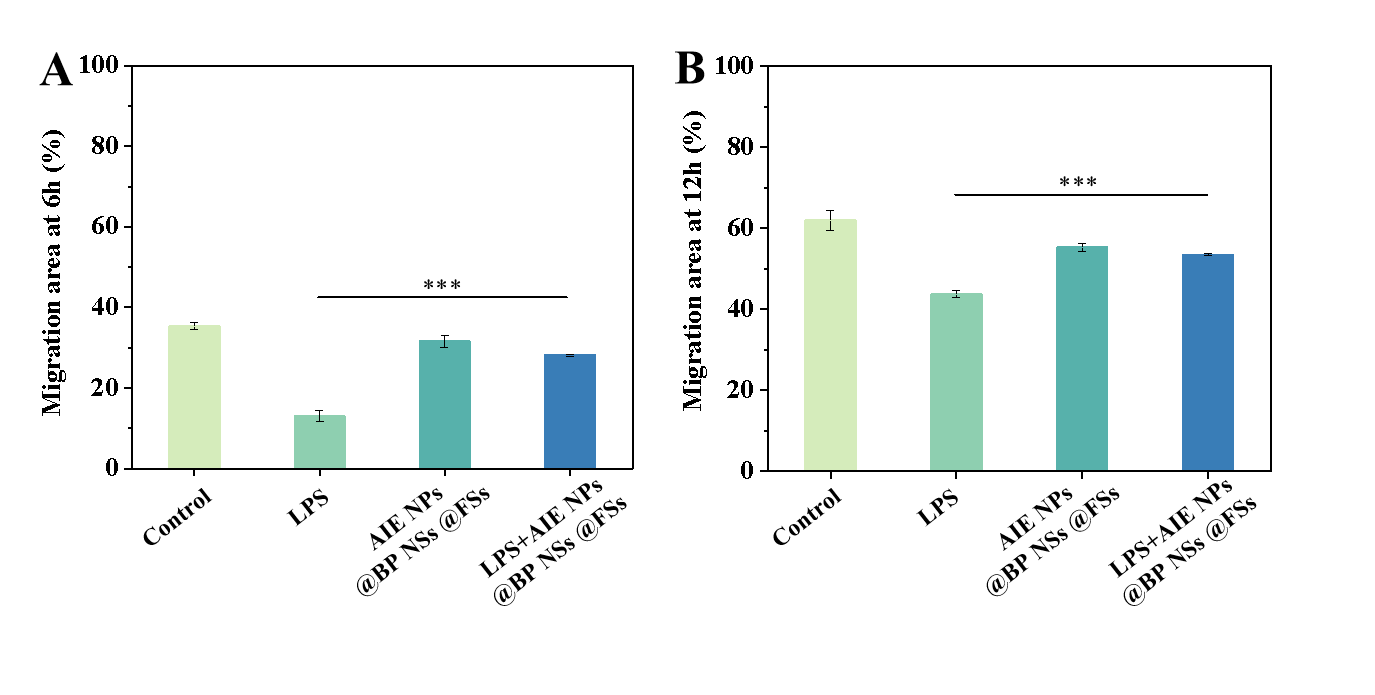


**Figure S31.** Quantitative analysis of scratch migration of L929 cells under different treatments at (A) 6h, and (B) 12h. Data are shown as mean ± s.d. (statistical significances were calculated by *t*-test: ****p < 0.001*, N=3).


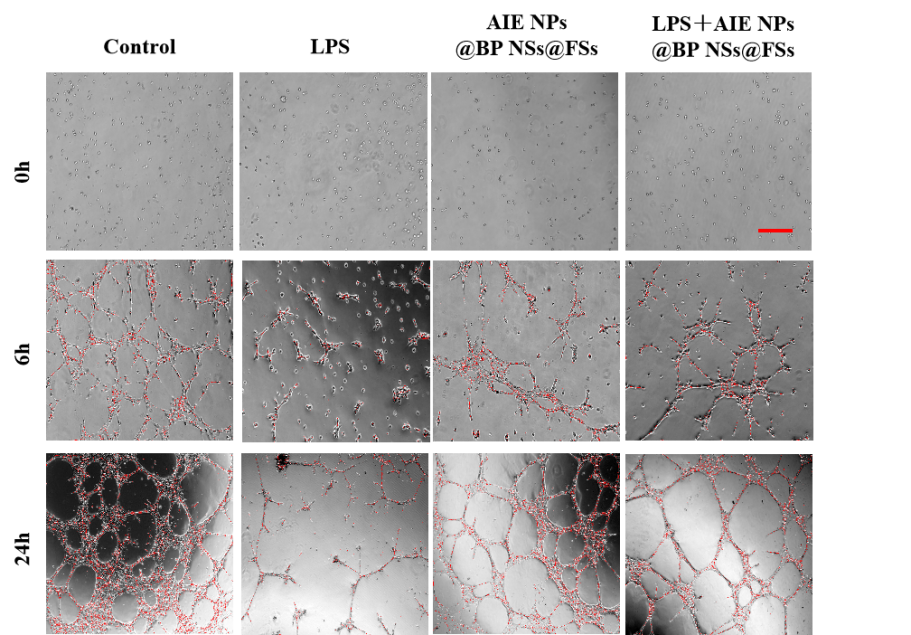


**Figure S32.** Representative images of the tube formation assay for MAECs under different treatments. Scale bar, 100 μm. Data are shown as mean ± s.d. (statistical significances were calculated by *t*-test: **p < 0.05, **p < 0.01, ***p < 0.001,* N=3).


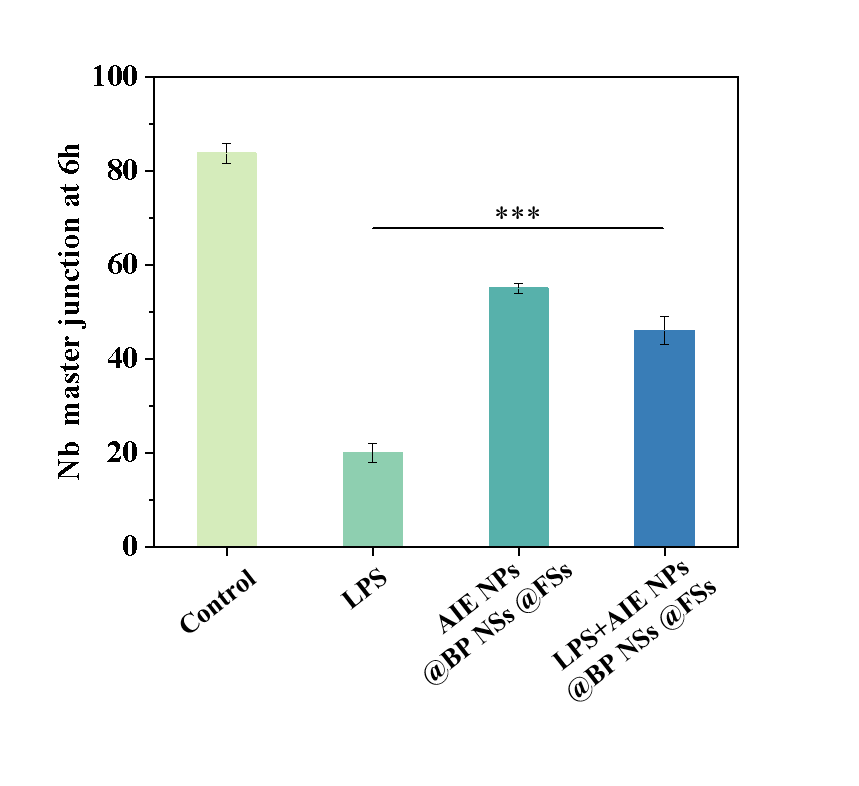


**Figure S33.** Quantitative analysis of the tube formation assay of MAECs under LPS and different treatments at 6h. Data are shown as mean ± s.d. (statistical significances were calculated by *t*-test: ****p < 0.001*, N=3).


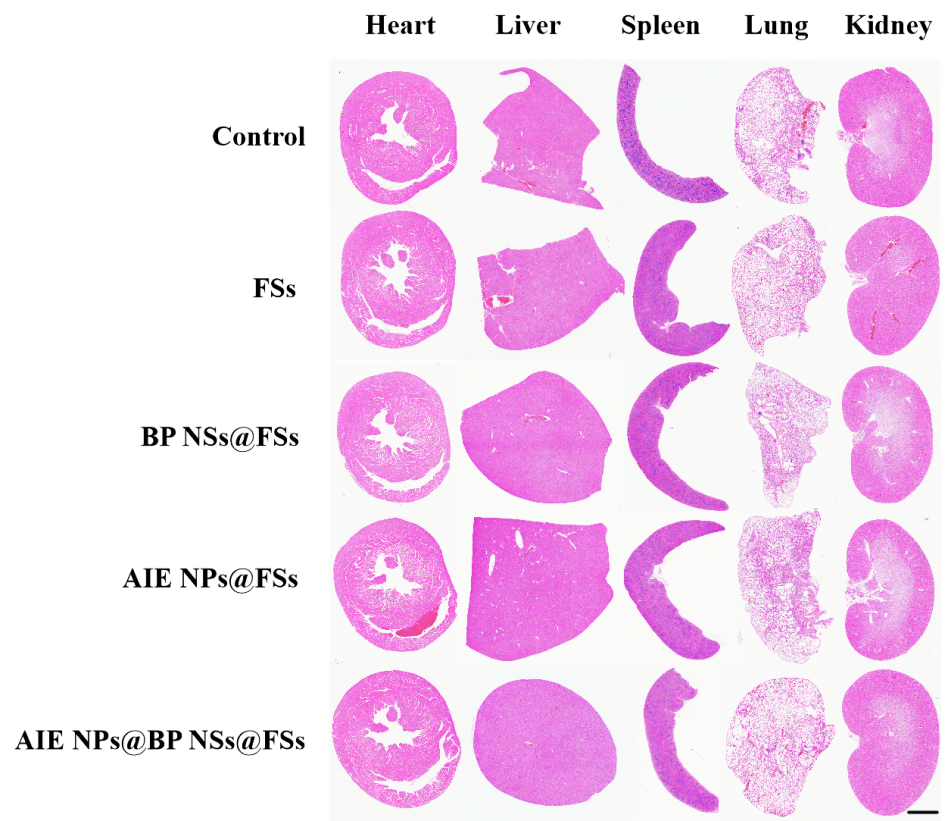


**Figure S34.** Histological staining of major organs in mice after different treatments. Scale bar, 50 mm.


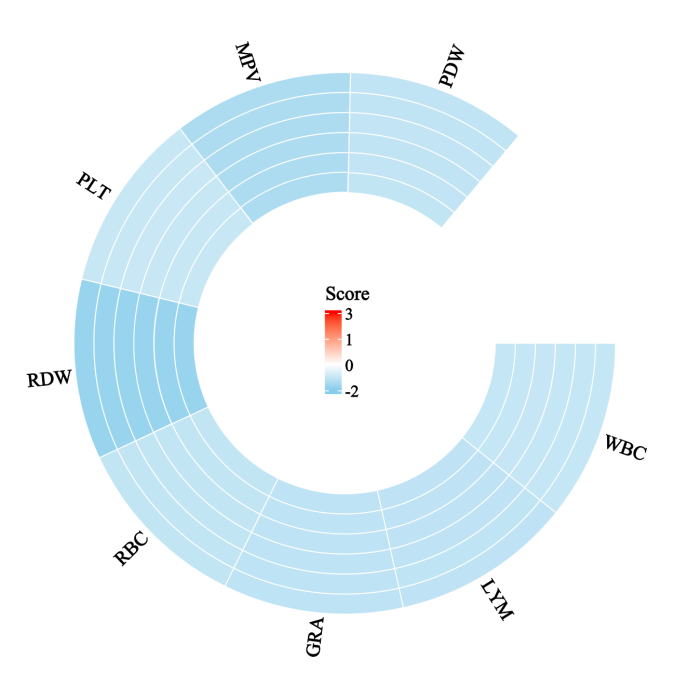


**Figure S35.** Heatmap analysis of hematological parameters (WBC, LYM, RBC, RDW, PLT, MPV, etc.) in the AIE NPs@BP NSs@FSs/NIR group and the control group.


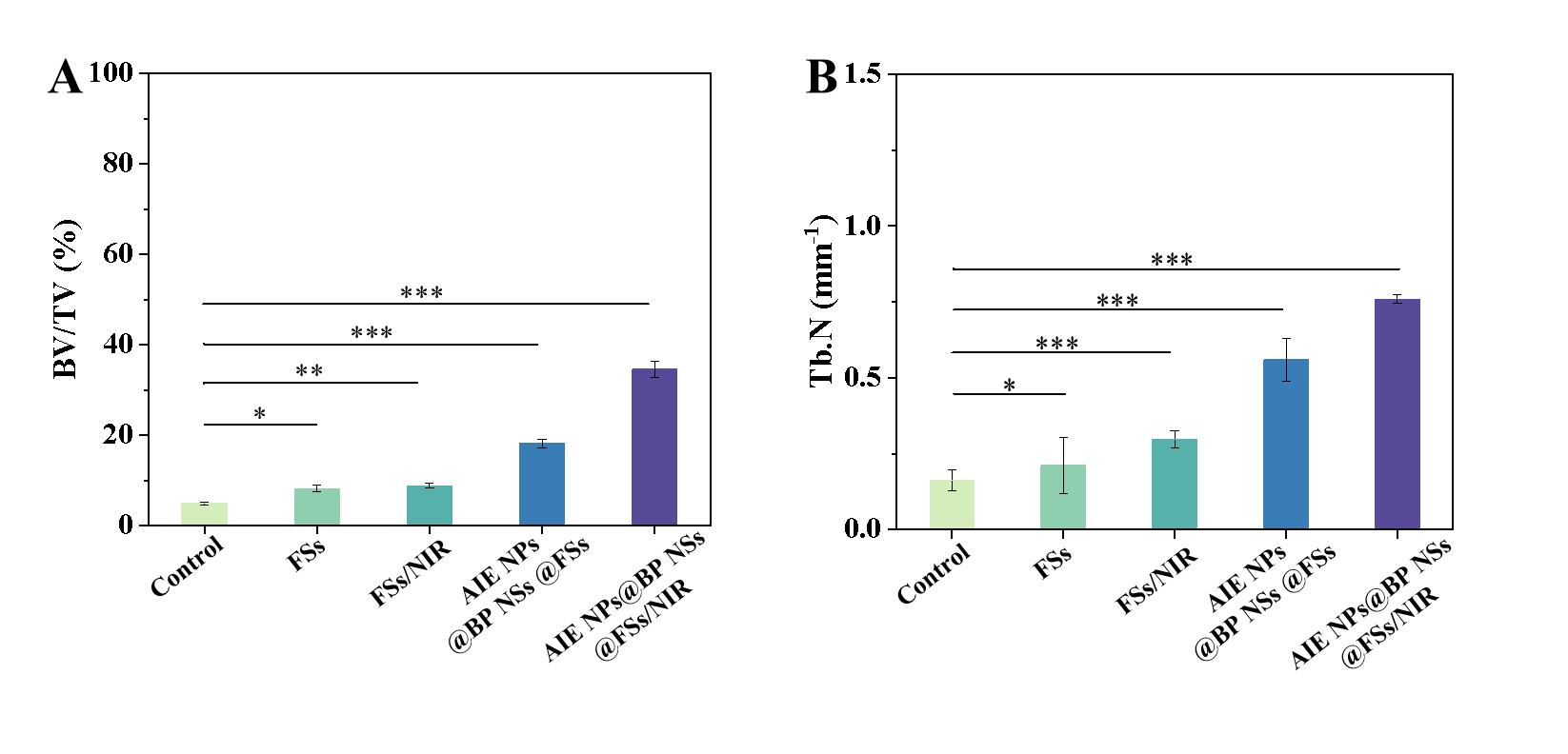


**Figure S36.** (A) Summarized data of BV/TV, and (B) Tb.N in the defect area, showing microarchitectural parameters of newly formed bone tissue at 4 weeks across various treatments. Data are shown as mean ± s.d. (statistical significances were calculated by *t*-test: **p < 0.05, **p < 0.01, ***p < 0.001,* N=3).


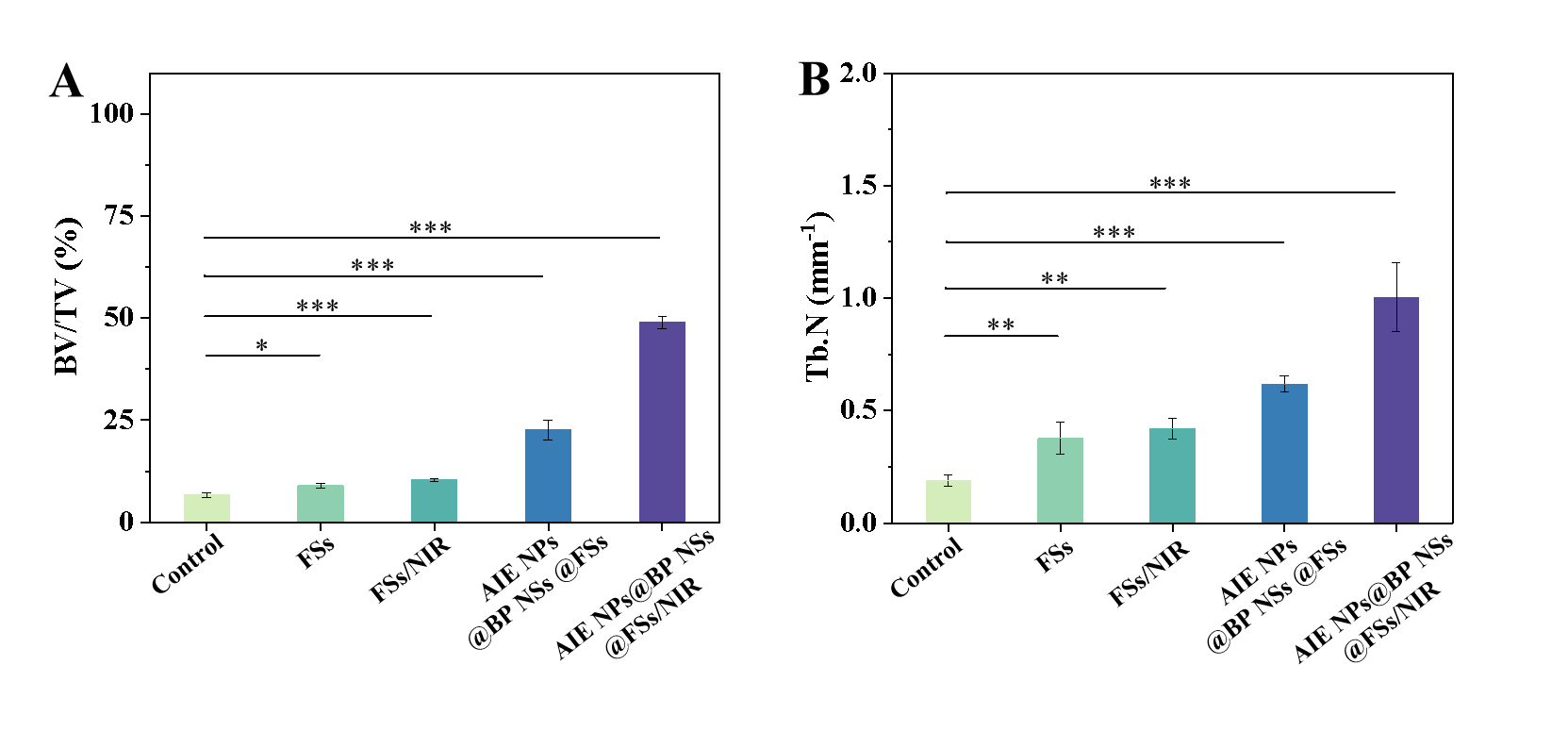


**Figure S37.** (A) Summarized data of BV/TV, and (B) Tb.N in the defect area, showing microarchitectural parameters of newly formed bone tissue at 8 weeks across various treatments. Data are shown as mean ± s.d. (statistical significances were calculated by *t*-test: **p < 0.05, **p < 0.01, ***p < 0.001,* N=3).


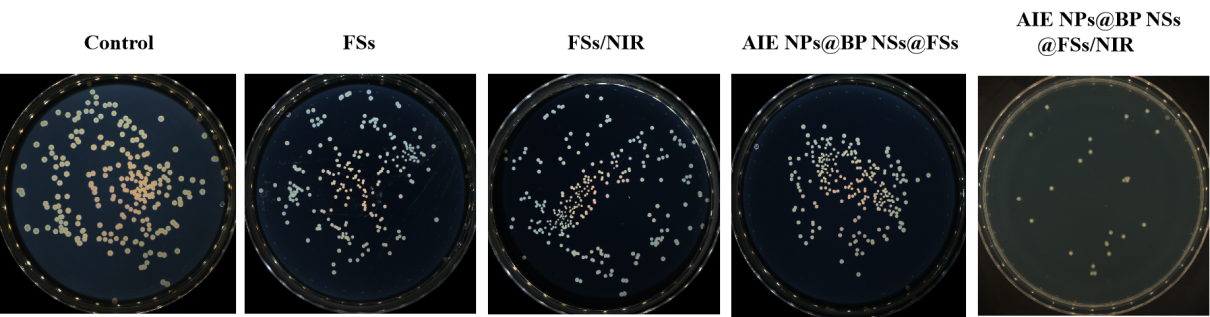


**Figure S38.** Images of the bacterial colonies cultured from the bone harvested 8 weeks post-treatment.


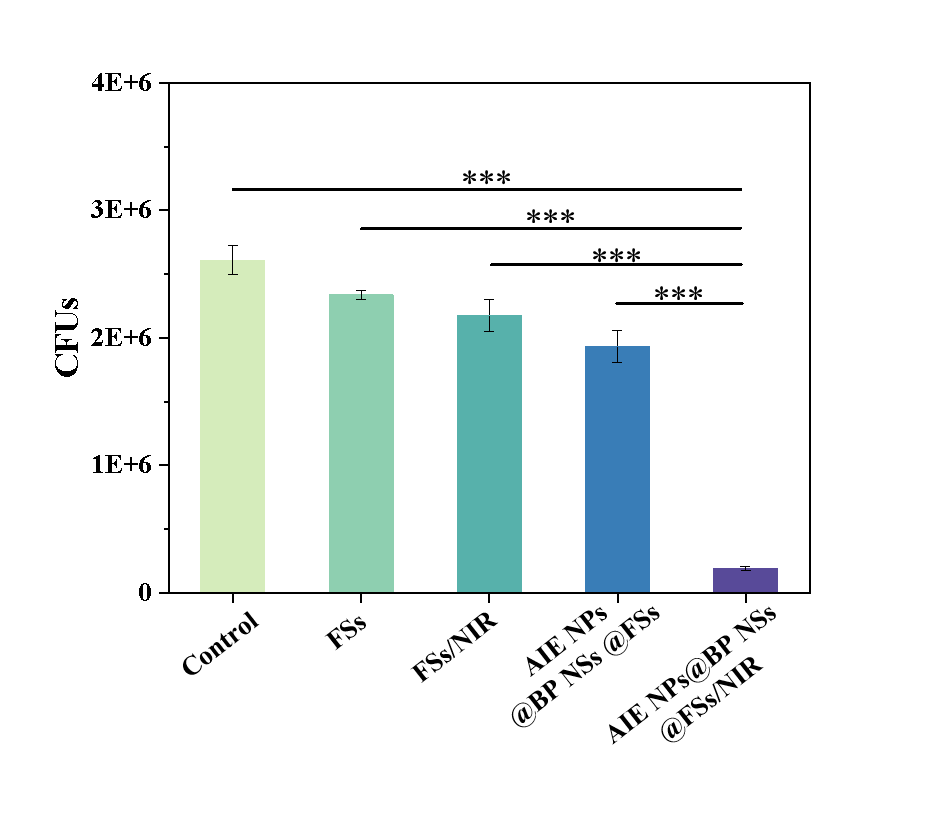


**Figure S39.** The bacterial colonies cultured from the bone were harvested 8 weeks post-treatment and quantification. Data are shown as mean ± s.d. (statistical significances were calculated by *t*-test: ****p < 0.001,* N=3).


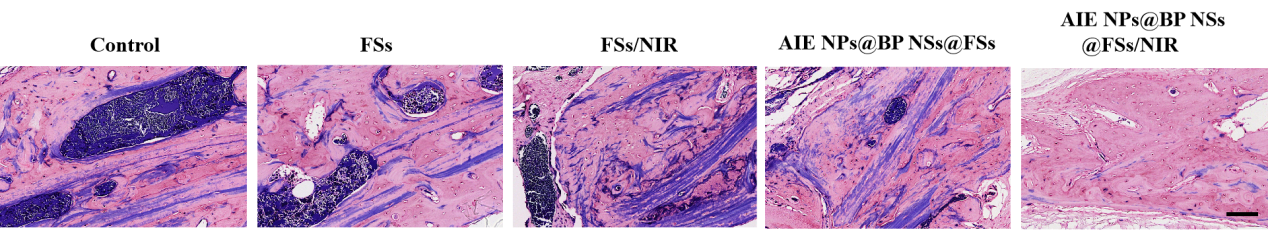


**Figure S40.** Tissue sections were Gram-stained at week 8 after treatment in each group. Scale bar, 100 μm.


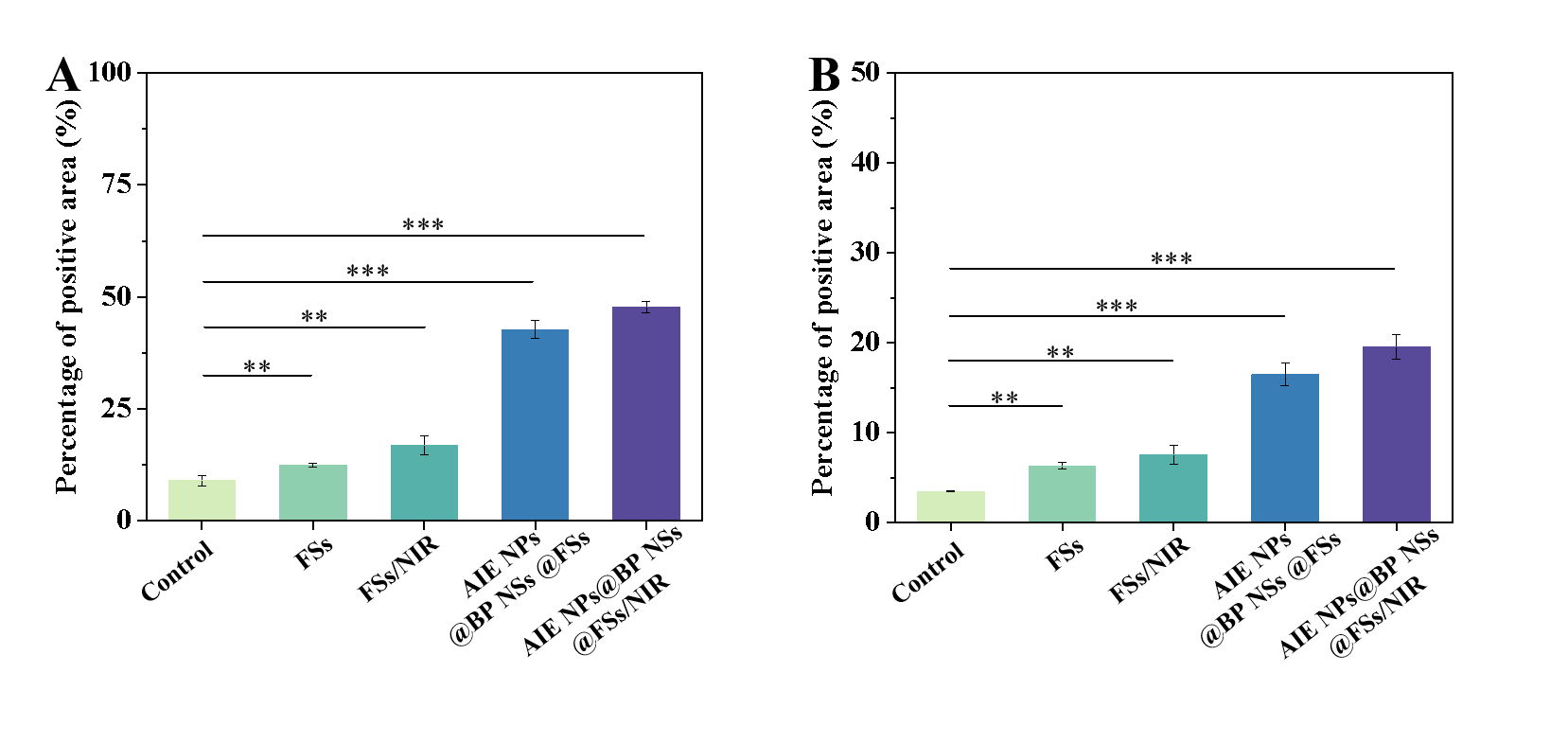


**Figure S41.** (A) Representative IHC staining of OCN after 8 weeks across various treatments, quantitative results of positive area. (B) Representative IHC staining of CD31 after 8 weeks across various treatments, and quantitative results of the positive area. Data are shown as mean ± s.d. (statistical significances were calculated by *t*-test: ***p < 0.01, ***p < 0.001,* N=3).


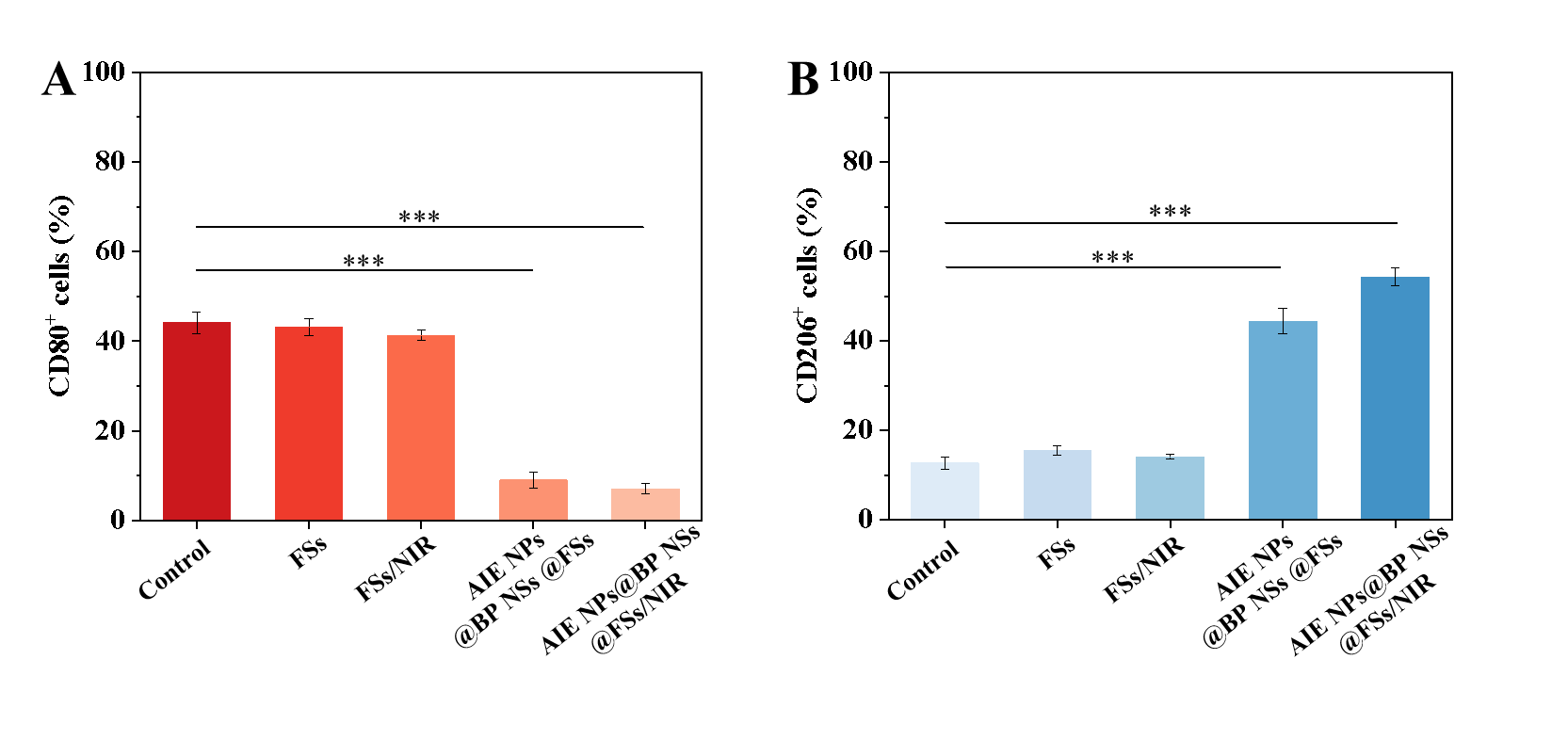


**Figure S42.** (A) Quantitative results of CD80^+^ fluorescence intensity in the bone defect region after 8 weeks of different treatments. (B) Quantitative results of CD206^+^ fluorescence intensity in the bone defect region after 8 weeks of different treatments. Data are shown as mean ± s.d. (statistical significances were calculated by *t*-test:  ****p < 0.001,* N=3).


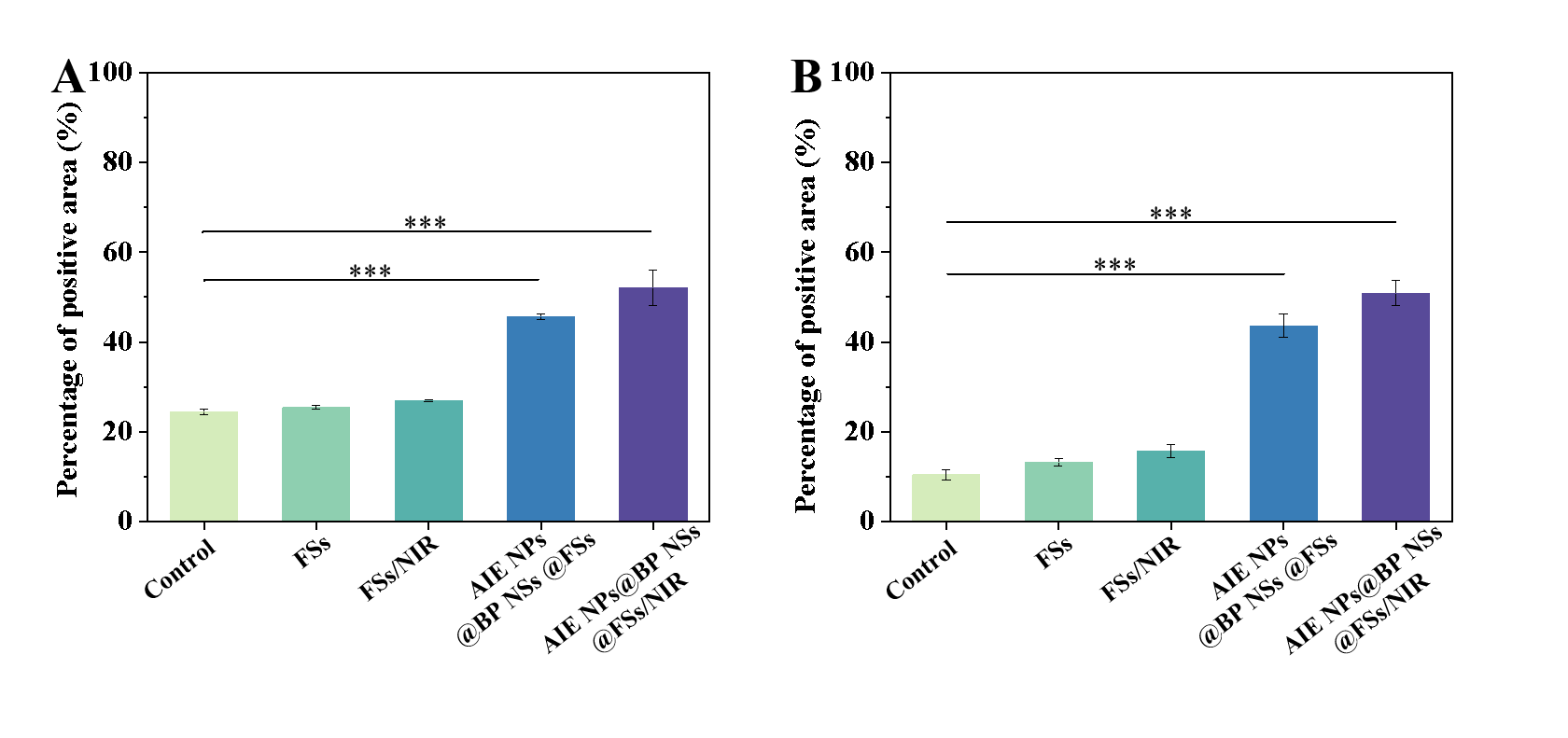


**Figure S43.** (A) Representative IHC staining of IL-10 after 8 weeks across various treatments, quantitative results of positive area. (B) Representative IHC staining of TGF-β after 8 weeks across various treatments, quantitative results of positive area. Data are shown as mean ± s.d. (statistical significances were calculated by *t*-test: ****p < 0.001,* N=3).


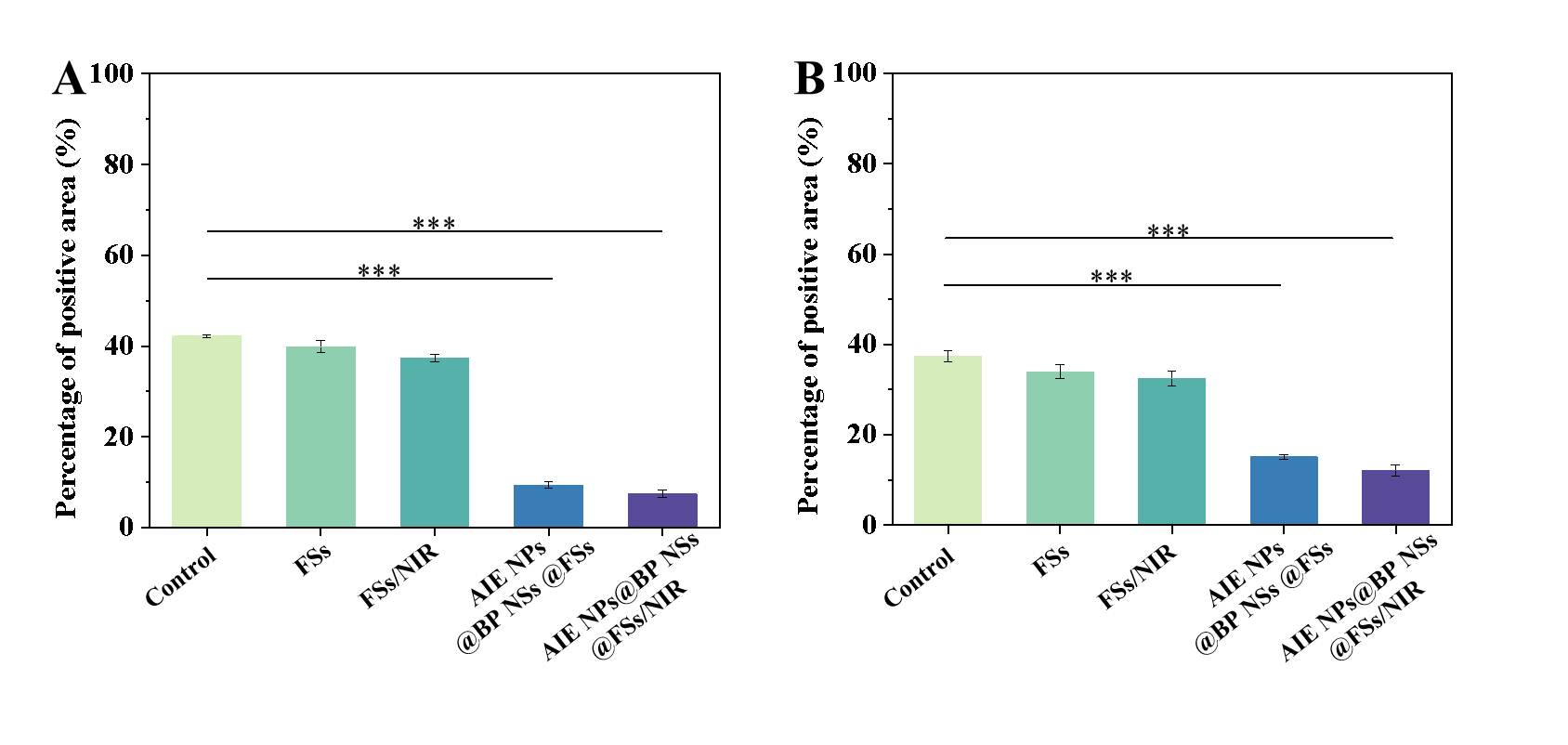


**Figure S44.** (A) Representative IHC staining of TNF-α after 8 weeks across various treatments, quantitative results of positive area. (B) Representative IHC staining of IFN-γ after 8 weeks across various treatments, with quantitative results of positive area. Data are shown as mean ± s.d. (statistical significances were calculated by *t*-test: ****p < 0.001,* N=3).


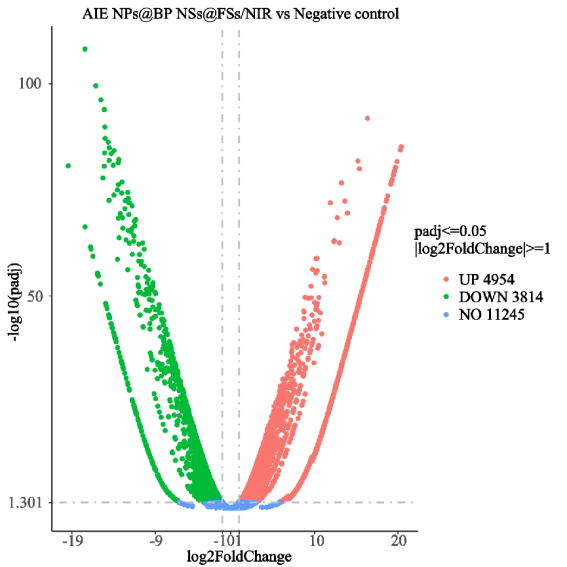


**Figure S45.** Volcano plot of differentially expressed genes following AIE NPs@BP NSs@FSs/NIR treatment.

**Supplementary tables**

**Table S1. Composition of electrospinning solution for membrane fabrication**

| Category | HFP (mL) | PCL (g) | Gel (g) | HAc (lL) | | AIE NPs (ml) | | BP NSs (ml) | |
| --- | --- | --- | --- | --- | --- | --- | --- | --- | --- |
| PCL/Gel  PCL/Gel/BP NSs  PCL/Gel/AIE NPs  PCL/Gel/AIE NPs/BP NSs | 15  15  15  15 | 1.2  1.2  1.2  1.2 | 0.3  0.3  0.3  0.3 | | 30  30  30  30 | | 0  4  0  4 | | 0  0  2  2 |

**Table S2.** Primer used for qPCR of *S. aureus*

| **Gene** | **Forward primers** | **Reverse primers** |
| --- | --- | --- |
| *sspA* | TGCCAACGATGACCAACCTA | CATTGTCTGGATTGTCTGGATTATC |
| *sspB* | GAAGCGATACAAGAAGATCAAGTTC | TAATGCTGCCATACTGAATCCTG |
| *arcC* | GGTATGATAGGCTATTGGTTGGAA | GGTTTGGTTGGGTTATTGAATCG |
| *arcD* | TTCTGCTGAATGACACCTTGG | TAGGAACTGCTACCGTTATTGG |
| *argH* | ACTTACCACCAGCATCACCAA | GCGAATCAAGGCATTATTAGTCAAC |
| *argF* | GATGGCATTGAATACCGTGGTT | CAGCAAGAACTTGAGTAGGATGAT |
| *16s* | GTCTTGCTGTCACTTATAGATGGA | GTTGCCTTGGTAAGCCGTTA |

**Table S3.** Primer used for qPCR of immune factors.

| **Gene** | **Forward primers** | **Reverse primers** |
| --- | --- | --- |
| *Tgf-β* | TGATACGCCTGAGTGGCTGTCT | CACAAGAGCAGTGAGCGCTGAA |
| *Il-10* | GAGAAGCATGGCCCAGAAATC | GAGAAATCGATGACAGCGCC |
| *Il-6* | CTGAACTTCGGGGTGATCGG | GGCTTGTCACTCGAATTTTGAGA |
| *Tnf-α* | GGTGCCTATGTCTCAGCCTCTT | GCCATAGAACTGATGAGAGGGAG |
| *Cd206* | AGACGAAATCCCTGCTACTG | CACCCATTCGAAGGCATTC |
| *Arg-1*  *Gapdh* | TGGAGAGTGTGGATCCCAAG  CATCATGCCACCCAGAAGACTG | GGTGCTGATGTACCAGTTGG  ATGCCAGTGAGCTTCCCGTTCAG |

**Table S4.** Primer used for qPCR of osteogenic factors.

| **Gene** | **Forward primers** | **Reverse primers** |
| --- | --- | --- |
| *Alpl* | TATGTCTGGAACCGCACTGAA | CACTAGCAAGAAGAAGCCTTT |
| *Runx2* | ATCCAGCCACCTTCACTTACAC | GGGACCATTGGGAACTGATA |
| *Bglap* | AACGGTGGTGCCATAGATGC | AGGACCCTCTCTCTGCTCAC |
| *Cal1a1* | CAGGCTGGTGTGATGGGATT | CCAAGGTCTCCAGGAACACC |
| *Spp1* | GCGGTTCACTTTGAGGACAC | TATGAGGCGGGGATAGTCTTT |
| *Gapdh* | GGCACGTCAAGGCTGAGAATG | ATGGTGGTGAAGACGCCAGTA |
